# Changes at a Critical Branchpoint in the Anthocyanin Biosynthetic Pathway Underlie the Blue to Orange Flower Color Transition in *Lysimachia arvensis*

**DOI:** 10.3389/fpls.2021.633979

**Published:** 2021-02-22

**Authors:** Mercedes Sánchez-Cabrera, Francisco Javier Jiménez-López, Eduardo Narbona, Montserrat Arista, Pedro L. Ortiz, Francisco J. Romero-Campero, Karolis Ramanauskas, Boris Igić, Amelia A. Fuller, Justen B. Whittall

**Affiliations:** ^1^Department of Plant Biology and Ecology, Faculty of Biology, University of Seville, Seville, Spain; ^2^Department of Molecular Biology and Biochemical Engineering, Pablo de Olavide University, Seville, Spain; ^3^Institute for Plant Biochemistry and Photosynthesis, University of Seville – Centro Superior de Investigación Científica, Seville, Spain; ^4^Department of Computer Science and Artificial Intelligence, University of Seville, Seville, Spain; ^5^Department of Biological Science, University of Illinois at Chicago, Chicago, IL, United States; ^6^Department of Chemistry and Biochemistry, Santa Clara University, Santa Clara, CA, United States; ^7^Department of Biology, College of Arts and Sciences, Santa Clara University, Santa Clara, CA, United States

**Keywords:** *DFR*, *F3′5′H*, flavonoid biosynthetic pathway, malvidin, pelargonidin, RNA-Seq

## Abstract

Anthocyanins are the primary pigments contributing to the variety of flower colors among angiosperms and are considered essential for survival and reproduction. Anthocyanins are members of the flavonoids, a broader class of secondary metabolites, of which there are numerous structural genes and regulators thereof. In western European populations of *Lysimachia arvensis*, there are blue- and orange-petaled individuals. The proportion of blue-flowered plants increases with temperature and daylength yet decreases with precipitation. Here, we performed a transcriptome analysis to characterize the coding sequences of a large group of flavonoid biosynthetic genes, examine their expression and compare our results to flavonoid biochemical analysis for blue and orange petals. Among a set of 140 structural and regulatory genes broadly representing the flavonoid biosynthetic pathway, we found 39 genes with significant differential expression including some that have previously been reported to be involved in similar flower color transitions. In particular, *F3′5′H* and *DFR*, two genes at a critical branchpoint in the ABP for determining flower color, showed differential expression. The expression results were complemented by careful examination of the SNPs that differentiate the two color types for these two critical genes. The decreased expression of *F3′5′H* in orange petals and differential expression of two distinct copies of *DFR*, which also exhibit amino acid changes in the color-determining substrate specificity region, strongly correlate with the blue to orange transition. Our biochemical analysis was consistent with the transcriptome data indicating that the shift from blue to orange petals is caused by a change from primarily malvidin to largely pelargonidin forms of anthocyanins. Overall, we have identified several flavonoid biosynthetic pathway loci likely involved in the shift in flower color in *L. arvensis* and even more loci that may represent the complex network of genetic and physiological consequences of this flower color polymorphism.

## Introduction

Flower color plays a fundamental role in pollinator attraction and plant reproduction (Fenster et al., 2004). Changes in flower color may produce pollinator shifts leading to speciation (Rausher, 2008; Ortiz-Barrientos, 2013; Van der Niet et al., 2014). Abiotic factors have received far less attention, but they may also impose selection on flower color (e.g., temperature, precipitation, solar radiation; Strauss and Whittall, 2006; Rausher, 2008; Van der Kooi et al., 2019; Dalrymple et al., 2020; Koski et al., 2020). The palette of flower color is diverse and there are a variety of mechanisms to control flower colors (cell shape, co-pigments, pH, etc.). However, large differences in flower color are mainly controlled by the underlying pigments such as chlorophylls, carotenoids, betalains and flavonoids (Tanaka et al., 2008). Anthocyanins are the most common group of flavonoid pigments present in flowers, and differences in the number of hydroxyl groups on their B-ring characterize the three main types: pelargonidin-derived anthocyanins (orange or red; one hydroxyl group), cyanidin-derived anthocyanins (magenta; two hydroxyl groups) and delphinidin-derived anthocyanins (including malvidin conferring blue or purple; three hydroxyl groups; Davies, 2009; Wessinger and Rausher, 2012, 2013; Ng et al., 2018).

Flower color variation exists both between and within species across angiosperms. Flower color transitions are common between related species (Zufall and Rausher, 2004; Tripp and Manos, 2008; Streisfeld and Rausher, 2009; Smith and Rausher, 2011; Wessinger and Rausher, 2013; Roberts and Roalson, 2017). However, some lineages exhibit striking flower color variation within species (Gigord et al., 2001; Schemske and Bierzychudek, 2007; Gómez et al., 2020). Flower color polymorphisms, the coexistence of two or more discrete flower color phenotypes within a population (Huxley, 1955; Schemske and Bierzychudek, 2007), is relatively common and concentrated in some families of angiosperms (Narbona et al., 2018). Flower color polymorphisms are frequently caused by the loss of anthocyanins (i.e., polymorphisms usually involving white-flowered plants) as in the cases of *Ipomoea purpurea* (purple and white; Rausher and Simms, 1989), *Linanthus parryae* (blue and white; Schemske and Bierzychudek, 2007), *Ruellia simplex* (purple, pink, and white; Freyre et al., 2015), *Parrya nudicaulis* (purple and white; Dick et al., 2011), *Raphanus sativus* (pink, bronze, yellow and white; Irwin and Strauss, 2005) and *Primula vulgaris* (purple, blue, pink, white, and yellow; Shipunov et al., 2011). Less commonly, flower color polymorphisms are caused by changes in anthocyanin composition as in *Ipomoea nil* (blue, magenta, purple, and red; Morita et al., 2005), *Phlox drummondii* (blue and red; Hopkins and Rausher, 2011) and *Lysimachia arvensis* (blue and orange; Arista et al., 2013). Streisfeld and Rausher (2011) predicted that evolutionary transitions in floral color are more likely caused by regulatory mutations in ABP genes instead of mutations in the coding sequence (CDS), because regulatory changes can be tissue-specific and are thus less likely to have deleterious pleiotropic effects. In this study, we focus on the flavonoid genes and compounds underlying the blue and orange flower color variation of *Lysimachia arvensis*.

Anthocyanins are synthesized by the anthocyanin biosynthetic pathway (ABP) – a highly conserved pathway among angiosperms and one of the best studied pathways for the production of secondary metabolites in plants (Holton and Cornish, 1995; Davies and Schwinn, 2006; Ho and Smith, 2016; Wheeler and Smith, 2019). The ABP is part of the flavonoid biosynthetic pathway, a broader metabolic pathway emerging from phenylpropanoid metabolism that includes flavone, flavonol, and anthocyanin synthesis plus the post-translational modifying steps like acylation and glycosylation (Clegg and Durbin, 2000; Sobel and Streisfeld, 2013; Chen et al., 2018; Gao et al., 2019). Approximately 100 structural genes (KEGG pathway database^1^) are involved in this broad flavonoid biosynthetic pathway. In addition, tissue-specific transcription factors play an important role in this pathway (Quattrocchio et al., 2006; Zhao et al., 2013), regulating those structural genes through the MYB-bHLH-WDR complex (MBW; Xu et al., 2015; Gao et al., 2019). This regulatory complex is conserved among vascular plants; however, the structural genes that they regulate varies among angiosperm lineages (Hichri et al., 2011; Lai et al., 2013; Albert et al., 2014; Xu et al., 2015). Regulatory complexity (e.g., tissue-specific regulation) is conferred by the large gene families for these proteins (e.g., R2R3 MYBs; Stracke et al., 2001; WD40; Xu et al., 2015). In particular, MYB regulators tend to be disproportionately involved in floral anthocyanin variation both within and between species.

The number of studies describing the causes of flower color variation in the wild is rapidly growing (e.g., Dick et al., 2011; Yuan et al., 2013; Casimiro-Soriguer et al., 2016; Kellenberger et al., 2019). Wheeler and Smith (2019) reviewed 15 studies involving natural evolutionary transitions between anthocyanin pigment types in which all 15 examples involved decreased expression in either *F3′H* or *F3′5′H*, or both genes (Wessinger and Rausher, 2012; Wheeler and Smith, 2019). Furthermore, two of these cases involved *DFR* mutations conferring substrate specificity, a secondary modification to redirect the flux directly down the pelargonidin branch of the ABP (Johnson et al., 2001; Zufall and Rausher, 2004; Smith and Rausher, 2011; Wheeler and Smith, 2019). According to Streisfeld and Rausher (2011), flower color variation associated with anthocyanin loss is more likely to be caused by regulatory genes mutations to avoid deleterious pleiotropic effects, while flower color variation associated with changes in anthocyanin type are more likely to be caused by mutations in structural genes, however Wheeler and Smith’s (2019) findings suggest both regulatory and structural changes are often correlated with the blue to red transition in natural flower color changes. Traditional approaches to finding the genes underlying flower color variation involve sequencing and determining expression in a relatively narrow group of candidate genes in the ABP (Le Maitre et al., 2019) which we will refer to as the narrow ABP. Alternatively, transcriptomic approaches using RNA-Seq allow a much broader investigation of the underlying causes of flower color variation (e.g., across the broader flavonoid biosynthetic pathway). For non-model species, *de novo* transcriptome assembly can be used to estimate genes with significant differential expression (DEGs; Wang et al., 2009; Martin and Wang, 2011; Grabherr et al., 2011; Anders et al., 2013; Haas et al., 2013; Butler et al., 2014; Casimiro-Soriguer et al., 2016; Chen et al., 2018; Duan et al., 2020). Even using a transcriptome approach, most studies of anthocyanin loss have focused on a narrow group of ABP candidate genes (Lulin et al., 2012; Lou et al., 2014; Casimiro-Soriguer et al., 2016; Gao et al., 2016; Lang et al., 2019; Li L. et al., 2019; Duan et al., 2020; Zhang et al., 2020). Herein, we describe the *de novo* assembled transcriptome for blue- and orange-flowered *Lysimachia arvensis* to determine differentially expressed genes among the broader flavonoid biosynthetic pathway.

*Lysimachia arvensis* (L.) Manns & Anderb. (Primulaceae), formerly *Anagallis arvensis*, is a self-incompatible (Gibbs and Talavera, 2001), tetraploid (2n = 40; Pujadas, 1997; Moneim et al., 2003) annual species displaying a petal color polymorphism. Blue- and orange-flowered plants, traditionally described as blue- and red-flowered plants (Ferguson, 1972), occur in monomorphic and polymorphic populations across its native range in Europe (Arista et al., 2013; Ortiz et al., 2015). The blue petals of *L. arvensis* contain malvidin derivatives, whereas orange petals contain pelargonidin derivatives (Lawrence et al., 1939; Harborne, 1968; Ishikura, 1981). Although both *L. arvensis* flower colors have long been described as different morphs, recent molecular phylogenetic studies indicate that these flower color phenotypes reflect very closely related, yet distinct, evolutionary lineages (Jiménez-López, 2019; Jiménez-López et al., in review) with some degree of reproductive isolation (Jiménez-López et al., 2020). Pollinators (mainly *Halictus* spp. bees; Ortiz et al., 2015; Jiménez-López et al., 2019) exhibit a preference for the blue individuals when given a choice. Across Europe, there is a latitudinal cline in flower color from north to south. Completely orange populations are common in the more mesic temperate latitudes of northern and central Europe whereas completely blue populations are most common in the more xeric Mediterranean environments of southern Europe with polymorphic populations in between (Arista et al., 2013). In combination with the ecological divergence of these two flower color variants across the species range, this case of incipient speciation provides an opportunity to investigate the role of the flavonoid biosynthetic pathway during speciation (Coyne and Orr, 1998; Widmer et al., 2009; Reiseberg and Blackman, 2010).

In this study, we aim to identify the underlying genetic and biochemical causes of flower color variation in *L. arvensis*. Previous studies have found that gene expression and/or gene structure at a critical branch-point in the anthocyanin biosynthetic pathway involving two genes, *F3′5′H* and *DFR*, are involved in most blue to red/orange flower color shifts (malvidin/cyanidin to pelargonidin; Davies, 2009; Streisfeld and Rausher, 2009, 2011; Smith et al., 2012; Ho and Smith, 2016; Wheeler and Smith, 2019). In order to identify the underlying genetic causes of petal color polymorphism in *L. arvensi*s, we performed *de novo* transcriptome assembly from RNA-Seq data. We then tested for differential expression across a broad panel of 94 structural and 46 regulatory genes involved in the flavonoid biosynthetic pathway. We also examined these two candidate genes for non-synonymous single nucleotide polymorphisms (SNPs) fixed between blue and orange flowers in reference to the known functional domains as described for other species (Johnson et al., 2001; Seitz et al., 2007; Smith et al., 2012; Guo et al., 2019). Finally, we interpret our transcriptome results in light of the flavonoid biochemical profiles of both flower colors using Ultra-High Performance Liquid Chromatography (UHPLC-MS).

## Materials and Methods

### Plant Material

For transcriptomic analysis, we collected seeds from blue-flowered plants that belong to a primarily blue-flowered population (blue:orange ratio = 10:1; Spain, Andalusia, Hinojos; 37°17′37″ N, 6°25′15″ W) and orange-flowered plants that belong to a monomorphic orange population (France, Corsica, Solenzara; 41°51′19″ N, 9°21′16″ E). Voucher specimens were deposited in the University of Seville Herbarium (Herbarium Numbers: SEV286467 and SEV279123). Although there are benefits from studying a mixed population in order to control for differences in genetic background that can arise due to geographic separation and/or local adaptation in allopatry, we used these two populations because we previously obtained four generations of inbred lines that exhibit reduced heterozygosity, which facilitates transcriptome assembly and analysis. These seeds were sown in a glasshouse at Santa Clara University (CA, United States), where the RNA extractions of blue- and orange-flowered samples were performed on the same days, alternating the preparation of samples between flower colors, in relatively homogeneous environmental conditions. We sampled all five petals from ∼30 flowers per plant, from eight blue- and eight orange-flowered plants. We avoided the “bullseye” at the base of each petal because this study is focused on the blue and orange flower color polymorphism, and we have no evidence that the bullseye is different between morphs based on visible and UV digital images (Supplementary Figure 1). All samples were taken from first-day anthesis flowers. Immediately after excision, petals were placed in cryotubes that were immediately immersed in liquid nitrogen and stored at −80°C to avoid subsequent changes in gene expression and RNA degradation. For flavonoid profiling, we sampled one blue and one orange plant from Spain and confirmed with 13 blue and 10 orange glasshouse-grown introduced plants from California. Each sample was composed of between 10 and 15 petals without the central bullseye. Petal samples were stored in microtubes containing 200 μl of MeOH with 1% HCl.

### RNA Extraction and *de novo* Assembly

Petals were homogenized using a mortar and pestle. Total RNA was then extracted following the Qiagen RNeasy^®^Plant Mini Kit protocol (Qiagen, Germany) with the addition of PEG 20,000 mol. wt. (550 μl, 2%; Gehrig et al., 2000) before the first filtering step. The addition of PEG was essential to achieve reasonable RNA concentrations for library preparation and sequencing. RNA samples were stored at −80°C until further analysis. RNA concentration and purity was initially assessed with a Nanodrop Nd-1000 (Thermo Fisher Scientific) and agarose gel, and then confirmed with a Bioanalyzer (Agilent, Santa Clara, CA, United States) before sequencing.

Individual libraries were barcoded, multiplexed and sequenced as 150 bp paired-end reads using two lanes on an Illumina Hi-Seq 2000 (Illumina, San Diego, CA, United States) through Novogene (Beijing, China). Raw paired-end Illumina reads were assessed for quality using FastQC (Andrews, 2010) and were processed using Rcorrector v1.0.4 (Song and Florea, 2015) to correct random sequencing errors. Then, reads were trimmed with Trimmomatic v0.39 (Bolger et al., 2014) to remove any read containing bases with Phred scores lower than 20, low quality reads less than 50 bp long, and any adapter or other Illumina-specific sequences that were still present. The remaining reads were filtered with Kraken 2 (Wood et al., 2019) to remove Small and Large Subunit ribosomal RNA (SILVA database; Quast et al., 2013) and contaminating reads (minikraken2_v2 database). Additionally, we used custom-built databases, derived from RefSeq libraries: UniVec_Core, viral, mitochondrion, plastid, plasmid, archaea, bacteria, protozoa, human and fungi. Filtered reads were combined across all samples into a single RNA-Seq data set. We conducted a *de novo* transcriptome assembly using Trinity *v2.8.5* (Grabherr et al., 2011) to generate a single reference transcriptome assembly for *L. arvensis*. Statistical information on the assembly quality was obtained with the “TrinityStats.pl” commands in the Trinity package and Bowtie2 software (Langmead and Salzberg, 2012). Trinity produces assembled genes and isotigs (which may represent alleles, isoforms, and/or splice variants). We used the assembled genes for the DEGs in order to have a single locus per expression measure and isotigs for the SNP and phylogenetic analysis allowing a balance of certainty in the assembly quality while maximizing ortholog coverage for phylogenetic inference.

### Identification of Differentially Expressed Genes (DEGs)

Since the *de novo* transcriptome assembly was done using the entire dataset as a single sample (blue- and orange-flowered plants combined), we then mapped the reads from each sample back onto the reference transcriptome. Trimmed mean of M-values (TMM, mean of log-expression ratios; Robinson and Oshlack, 2010), were calculated for each gene with RSEM software (Li and Dewey, 2011) using a Pearl script (*align_and_estimate_abundance.pl*) provided with the Trinity distribution. Then, we used EdgeR package (Robinson et al., 2009) in R v4.0.0 (R Core Team, 2020) to determine statistically significant differentially expressed genes (DEGs) between different colored samples. In order to be conservative in DEG identification, we set two thresholds for determining significant differential expression: the false discovery rate (FDR) was less than 10^–5^ and the expression difference threshold was greater than one log_2_ fold-change (log_2_FC). Wilcoxon rank-sum test for ABP and non-ABP gene comparisons were conducted in R v4.0.0 (R Core Team, 2020).

In order to focus on a broadly defined set of genes associated with the broader flavonoid biosynthetic pathway, we explored 94 structural loci (Supplementary Table 1). These genes start at the phenylpropanoid biosynthetic pathway and continue through the entire flavonoid biosynthetic pathway to include the ABP and three non-anthocyanin flavonoid side-branches: flavone, flavonol and isoflavonoid biosynthetic pathways (KEGG pathway database^1^). In addition, we selected 46 regulatory genes involved in flavonoid pathway regulation, including the gene families for R2R3-Myb, bHLH and WD40 loci (Li, 2014; Xu et al., 2015; Gates et al., 2017; Supplementary Table 2).

To identify and annotate the flavonoid biosynthetic pathway genes in our *de novo* transcriptome assembly, we used Kakapo^2^. As input, we specified a list of 140 Genbank accessions for the genes involved in flavonoid biosynthetic pathway, restricted to the Order Ericales (if the gene was not found within Ericales, we removed the Order restriction).

### Phylogenetic Analysis of *F3′5′H* and *DFR*

Based on previous studies and our expression results, we carefully examined the RNA sequences of *F3′5′H* and *DFR* in relation to a set of Genbank reference sequences. In order to generate sequences of the coding region for these two enzymes, we mapped the Illumina reads of every sample to each *F3′5′H* or *DFR* isotig that arose from our Trinity *de novo* assembly. We used the “Map to Reference” function in Geneious v9.0.4 (Kearse et al., 2012), applying “highest sensitivity/slow” and “trim sequences” parameters. A subset of these isotigs, across orange- and blue-flowered samples for each gene, were used as queries in BLASTn tool from NCBI^3^ to locate homologous loci from a group of reference sequences (see results section). We selected a set of Genbank references that represented the top hits and some of the best-studied model systems in flower color research for our phylogenetic analysis. For each gene, the isotigs and Genbank reference sequences were aligned using the MUSCLE plug-in from within Geneious v9.0.4. Isotigs missing large portions of the coding sequence or containing >30% ambiguities were removed from the alignment, thus excluding some putative splice variants to improve accuracy. For *DFR*, we removed the 5′ and 3′ ends (24 and 52 bp, respectively) of the coding sequence (CDS) due to alignment ambiguities before conducting phylogenetic analysis. We reconstructed the relationships using the RAxML 7.2.8 plug-in from within Geneious v9.0.4, searching for the maximum likelihood tree with 100 bootstrap replicates. We then interpreted the phylogenetic tree in light of normalized expression values (TMM) in R v4.0.0 (R Core Team, 2020).

### SNP Frequencies for *F3′5′H* and *DFR*

In addition, we examined the frequency of non-synonymous SNPs that showed some tendency to differentiate flower color types for *F3′5′H* and *DFR-1* (*DFR-2* was only present in orange-flowered individuals so no sequences were available from blue-samples for comparison). For each sample, every isotig was determined to be fixed or variable. Although *L. arvensis* is tetraploid, we treated these as either homozygotes or heterozygotes (respectively) for our frequency calculations. We calculated the frequency of each non-synonymous SNP that showed differences between the flower color types providing BLOSUM62 scores (Henikoff and Henikoff, 1992), an empirical measure that estimates the biological probability of an amino acid substitution based on an alignment, and noting the biochemical properties of the amino acids involved. Furthermore, we extended this survey to include synonymous color-differentiating SNPs in the CDS for *F3′5′H* and *DFR-1*, including SNPs in the 5′ and 3′ UTRs for *F3′5′H*. In addition, since there were two copies of *DFR*, we also examined all non-synonymous amino acid substitutions between *DFR-1* and *DFR-2* in light of the location of variation in known functional domains responsible for substrate specificity and binding (Johnson et al., 2001; Petit et al., 2007; Smith et al., 2012). For both genes the amino acid residue numbers are based on the *L. arvensis* alignment, which translates to *Vitis vinifera* plus two amino acids (Petit et al., 2007).

### Identification of Flavonoid Compounds by UHPLC-MS

Flavonoids were extracted by placing 10–15 petals of blue and orange *L. arvensis* flowers in microtubes containing 200 μl of MeOH with 1% HCl. Homogenization was performed with a BeadBeater using 3 mm × 3.2 mm steel balls for 30 s. Samples were stored at −80°C until they could be loaded on a Dionex UltiMate 3000 ultra-high pressure liquid chromatography system equipped with a diode array detector and connected to a Thermo Fleet LCQ mass spectrometer (purchased from Thermo Fisher Scientific, Waltham, MA, United States). Data were analyzed using Xcalibur software (Thermo Fisher Scientific).

Crude extracts were eluted through a Phenomenex Gemini Column (50 mm × 2 mm, 3 micron; acquired from Phenomenex, Torrance, CA, United States) with a multi-step gradient of two solvents: 0.5% formic acid in water (solvent A) and 0.5% formic acid in methanol (solvent B) at a flow rate of 0.5 mL/min. The multi-step gradient involved an initial 2 min elution at 15% B, followed by a linear gradient over 5 min to 50% B, then isocratic elution at 50% B for 3 min, then flushing the column with 100% B for 3 min. The diode array detector collected UV-Vis spectra of eluted compounds from 190–700 nm. Mass spectra of eluted compounds were ionized using electrospray ionization and the mass detector was set to positive ion detection mode with a mass range of 150–800 Da.

Flavonoid identification was based on the compound’s retention time, UV-Vis spectra and whenever possible, chromatographic comparisons with authentic standards. We used flavonoid standards including quercetin, luteolin, kaempferol, isorhamnetin, malvidin, pelargonidin, cyanidin and delphinidin (purchased from Extrasynthese, Genay, France) that were previously reported for *L. arvensis* and other *Lysimachia* species (Supplementary Table 3). Putative identifications were also compared with published flavonoid data^4^.

## Results

### RNA Extraction and *de novo* Assembly

Bioanalyzer results confirmed we extracted high-quality RNA without substantial degradation. All RNA integrity numbers (RIN) were above the recommended RIN = 6.3 (Novogene, unpublished). The mean RIN value was 8.0 (6.7–9.0). The total number of filtered reads in the transcriptome analysis ranged from 21 million to 41 million in blue samples (mean of 26 million) and from 21 million to 26 million in orange samples (mean of 24 million). Approximately 98% of filtered reads of each sample mapped to the *de novo* assembled *L. arvensis* reference transcriptome. Isotig number per sample varied from 146 thousand to 193 thousand and the number of genes ranged from 37 thousand to 42 thousand (Supplementary Table 4). The GC percentage of the assembly was 38.27% and it had an N50 value of 1,635 bp. The average contig length was 973.5 bp and the total number of assembled bases equaled 389,888,932 bp. A total of 189,189 trinity genes and 400,523 trinity isotigs were obtained.

### Identification of Differentially Expressed Genes (DEGs)

Initially, we identified 48,306 differentially expressed genes and 102,750 differentially expressed isotigs in the *L. arvensis* transcriptome with EdgeR. After applying the cut-off values for FDR and log_2_FC, we reduced the number of DEGs to 10,624 genes. There were 6,966 DEGs with higher expression in blue samples and 3,658 DEGs with higher expression in orange samples (Supplementary Figure 2). The average log_2_FC of DEGs for blue > orange (B > O) was 2.39 and for O > B was 3.16.

**FIGURE 1:**
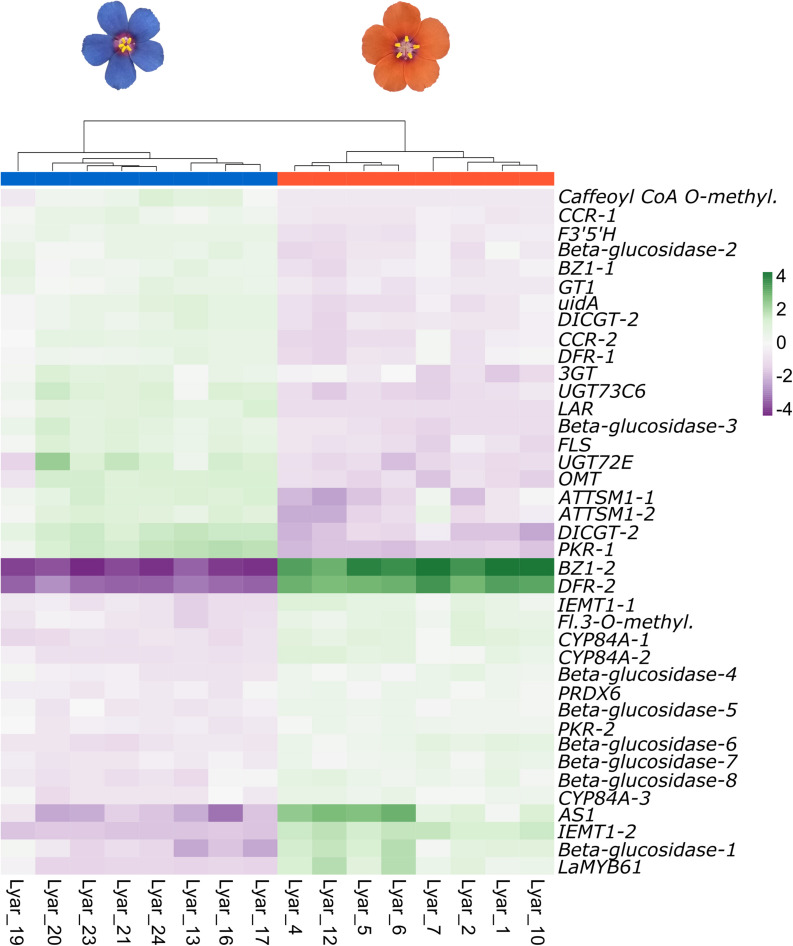
Clustering and heatmap of differentially expressed genes (DEGs) from the flavonoid biosynthesis pathway in blue- and orange-flowered plants of *L. arvensis*. The color scale represents the scaled TMM expression values (trimmed mean of *M*-values). Green represents high expression and purple represents low expression.

**FIGURE 2:**
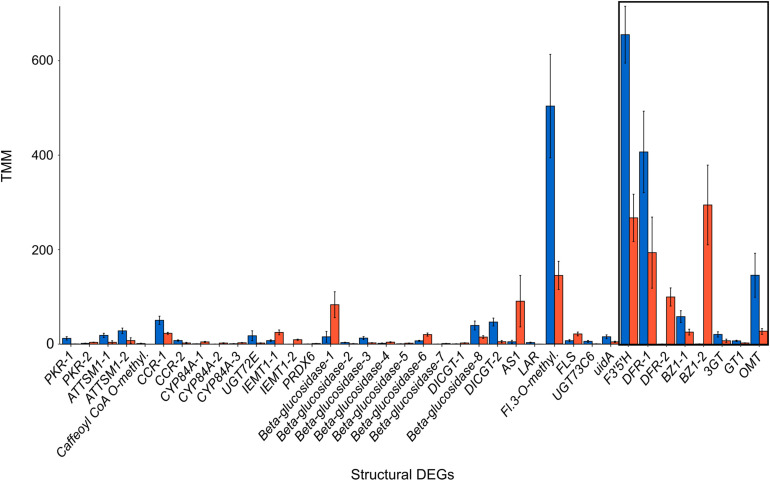
Expression of structural DEGs of the flavonoid biosynthesis pathway with significant differential expression between blue and orange *L. arvensis*. The colored bars represent the average expression level in TMM (trimmed mean of M-values) units and error bars show standard deviation. The black box indicates DEGs from the narrow ABP.

Restricting our analysis to the flavonoid biosynthetic pathway, our Kakapo results identified 60% (58/94) of the structural genes and 20% (10/46) of the regulatory genes in our 140 gene panel. Since some loci were found in multiple copies, a total of 153 structural gene copies and 13 regulatory gene copies were examined for differential expression (Supplementary Tables 5, 6; these tables contain the number of copies for each gene identified). Gene expression analysis showed 38 structural genes and one regulatory gene with significant differential expression between flower colors (Figure 1 and Table 1). Among the DEGs, 22 showed higher expression in blue-flowered plants, while 17 showed higher expression in orange-flowered plants (Figure 1). Structural DEGs from the early steps of the flavonoid biosynthetic pathway have lower expression levels (TMM) compared to DEGs from the narrow ABP (Wilcoxon rank-sum test, *W* = 46606, *p* = 2.2 × 10^–16^; Figure 2). These trend is also present within flower colors (orange ABP gene expression is 4.8× higher than orange non-ABP gene expression; Wilcoxon rank-sum test, *W* = 12111, *p* = 1.3 × 10^–12^; blue ABP gene expression is 6× higher than blue non-ABP gene expression; Wilcoxon rank-sum test, *W* = 11278, *p* = 8.5 × 10^–9^). In general, structural DEGs with higher expression in blue samples tended to have ∼2× higher overall expression than those genes with higher expression in orange samples (average TMM_*blue*_ = 55 vs. average TMM_*orange*_ = 29; Figure 2). Focusing on the narrow ABP, we obtained eight structural DEGs with higher expression in blue *L. arvensis* (Table 1 and Figure 2). On the contrary, we only found two with higher expression in orange *L. arvensis* (Table 1 and Figure 2). Two candidate genes involved in the blue to orange transition had significant differential expression (*F3′5′H* and two copies of *DFR*). *F3′5′H* and *DFR-1* show 2.5× and 2× B > O, respectively, while *DFR-2* has > 600× O > B (this locus is nearly undetectable in blue flowers). In regard to the regulatory genes, we found 13 trinity genes from four different gene families (Supplementary Figure 3 and Supplementary Table 6). Only *MYB61* (*MYB4-2*) showed significant differential expression with 26× higher expression in orange- than in blue-flowered plants (Figure 1 and Table 1). This MYB was initially identified as a *MYB4* from *Hibiscus syriacus* by Kakapo, but a BLASTn of this *L. arvensis* sequence showed that it is more similar to *Camellia sinensis MYB61*, and will be referred to as *LaMYB61* from here onward.

**TABLE 1:** Flavonoid biosynthetic pathway genes with significant differential expression between blue- and orange-flowered *L. arvensis*.

Gene abbreviation	Gene name	Expression in blue (TMM)	Expression in orange (TMM)	Log fold-change	Pathway*
*BZ1-2*	*Anthocyanin 3-O-glucosyltransferase*	0.31	294.55	−9.81	ABP
*DFR-2*	*Dihydroflavonol 4 reductase*	0.15	99.80	−9.29	ABP
*IEMT1-2*	*(Iso)eugenol O-methyltransferase*	0.05	9.34	−7.59	Non-ABP
*CYP84A-2*	*Ferulate 5-hydroxylase*	0.08	2.25	−4.74	Non-ABP
*LaMYB61*	*Transcription factor MYB61*	0.20	5.27	−4.68	Regulatory
*AS1*	*Aureusidin synthase*	4.98	90.89	−4.20	Non-ABP
*CYP84A-1*	*Ferulate 5-hydroxylase*	0.66	4.72	−2.84	Non-ABP
*Beta-glucosidase-7*	*Beta-glucosidase*	0.19	1.29	−2.69	Non-ABP
*Beta-glucosidase-1*	*Beta-glucosidase*	15.47	83.41	−2.45	Non-ABP
*Beta-glucosidase-8*	*Beta-glucosidase*	0.47	2.44	−2.42	Non-ABP
*PRDX6*	*Peroxiredoxin 6,1-Cys peroxiredoxin*	0.28	1.29	−2.17	Non-ABP
*IEMT1-1*	*(Iso)eugenol O-methyltransferase*	7.42	24.85	−1.77	Non-ABP
*CYP84A-3*	*Ferulate 5-hydroxylase*	0.87	2.84	−1.72	Non-ABP
*Beta-glucosidase-5*	*Beta-glucosidase*	0.66	2.01	−1.63	Non-ABP
*Fl. 3-O-methyl.*	*Flavonol 3-O-methyltransferase*	7.26	21.53	−1.59	Non-ABP
*Beta-glucosidase-6*	*Beta-glucosidase*	6.94	20.24	−1.56	Non-ABP
*Beta-glucosidase-4*	*Beta-glucosidase*	1.75	4.14	−1.26	Non-ABP
*PKR-2*	*Polyketide reductase*	1.66	3.80	−1.20	Non-ABP
*DFR-1*	*Dihydroflavonol 4 reductase*	406.80	193.69	1.06	ABP
*CCR-1*	*Cinnamoyl CoA reductase*	50.45	22.93	1.12	Non-ABP
*BZ1-1*	*Anthocyanin 3-O-glucosyltransferase*	58.20	25.46	1.18	ABP
*F3′5′H*	*Flavonoid 3′5′-hydroxylase*	655.06	267.30	1.28	ABP
*DICGT-1*	*Chalcononaringenin 2′-O-glucosyltransferase*	39.46	14.95	1.39	Non-ABP
*GT1*	*Anthocyanin 5,3-O-glucosyltransferase*	6.88	2.47	1.47	ABP
*3GT*	*Anthocyanin 3′-O-beta-glucosyltransferase*	20.51	7.14	1.50	ABP
*CCR-2*	*Cinnamoyl CoA reductase*	7.64	2.40	1.66	Non-ABP
*uidA*	*Beta glucuronidase*	15.59	4.82	1.68	Non-ABP
*FLS*	*Flavonol synthase*	503.38	144.86	1.77	Non-ABP
*ATTSM1-2*	*Caffeoyl CoA 3-O-methyltransferase*	28.12	7.53	1.89	Non-ABP
*Beta-glucosidase-2*	*Beta-glucosidase*	3.36	0.85	1.96	Non-ABP
*ATTSM1-1*	*Caffeoyl CoA 3-O-methyltransferase*	18.57	3.84	2.26	Non-ABP
*Beta-glucosidase-3*	*Beta-glucosidase*	12.76	2.62	2.26	Non-ABP
*OMT*	*O-methyltransferase*	145.81	27.40	2.39	ABP
*UGT72E*	*Coniferyl alcohol glucosyltransferase*	17.68	2.26	2.94	Non-ABP
*DICGT-2*	*Chalcononaringenin 2’-O-glucosyltransferase*	46.75	5.20	3.16	Non-ABP
*UGT73C6*	*Flavonol 3-O-L-rhamnoside-7-O-glucosyltransferase*	5.89	0.51	3.50	Non-ABP
*PKR-1*	*Polyketide reductase*	12.16	0.40	4.88	Non-ABP
*LAR*	*Leucoanthocyanidin reductase*	3.28	0.09	5.25	Non-ABP
*Caffeoyl CoA O-methyl.*	*Caffeoyl CoA-O-methyltransferase*	1.27	0.00	7.65	Non-ABP

Expression is calculated by the average TMM values per gene per each color. Negative Log fold-change values indicate a higher expression in orange samples, while positive values indicate higher expression in blue samples. *ABP: genes that belong to the narrow ABP; Non-ABP: genes that belong to the broader flavonoid biosynthetic pathway, excluding ABP genes; Regulatory genes indicated.

### Phylogenetic Analysis of *F3′5′H* and *DFR*

**FIGURE 3:**
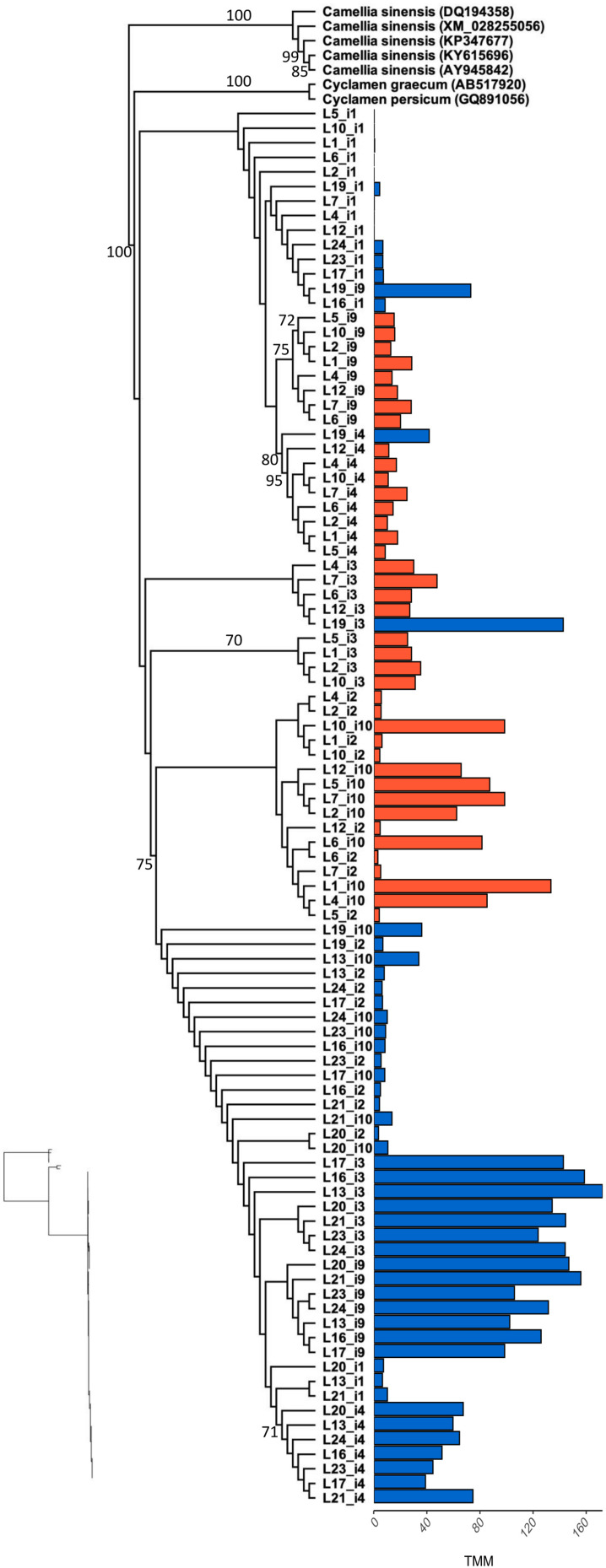
Maximum likelihood phylogenetic analysis of *F3′5′H* coding sequence for all *L. arvensis* isoforms (L1–12 = orange; L13–24 = blue). We included the top BLASTn hits for reference (scientific name followed by followed by Genbank Accession numbers). Bootstrap values are provided along the branch or at the node for values above 70%. The bar plot shows the expression value (TMM) per isotig for blue and orange flower colors. The isotigs with markedly shorter coding sequences than the Genbank references are *F3′5′H-i1* and *F3′5′H-i4*. The maximum likelihood phylogram without tip labels (yet same order) is included to allow for relative branchlength comparisons.

Our reference-guided assembly produced complete CDS for the 67% of *F3′5′H* isotigs and 30% of *DFR-1* isotigs, both in blue- and orange-flowered samples, while the 100% of *DFR-2* isotigs were exclusively identified in orange-flowered samples. The phylogenetic analysis of *F3′5′H* produced a tree with monophyletic clade of *L. arvensis* samples sister to *Cyclamen graecum* (Accession QG891056) and closely related to several *Camellia sinensis* samples (Figure 3). Isotigs cluster in monophyletic or nearly monophyletic clades for each flower color (e.g., orange-i2 and orange-i10 form a clade; a large clade with nearly all blue samples). There are no signs of gene duplication within *L. arvensis F3′5′H*. Sample L19 (blue) may be a hybrid between blue and orange (even though these are inbred lines) since for some isotigs, L19 clusters with orange samples (i1, i4, i3) and for other isotigs, it arises from the base of a clade of blue-flowered samples (i2, i10).

**FIGURE 4:**
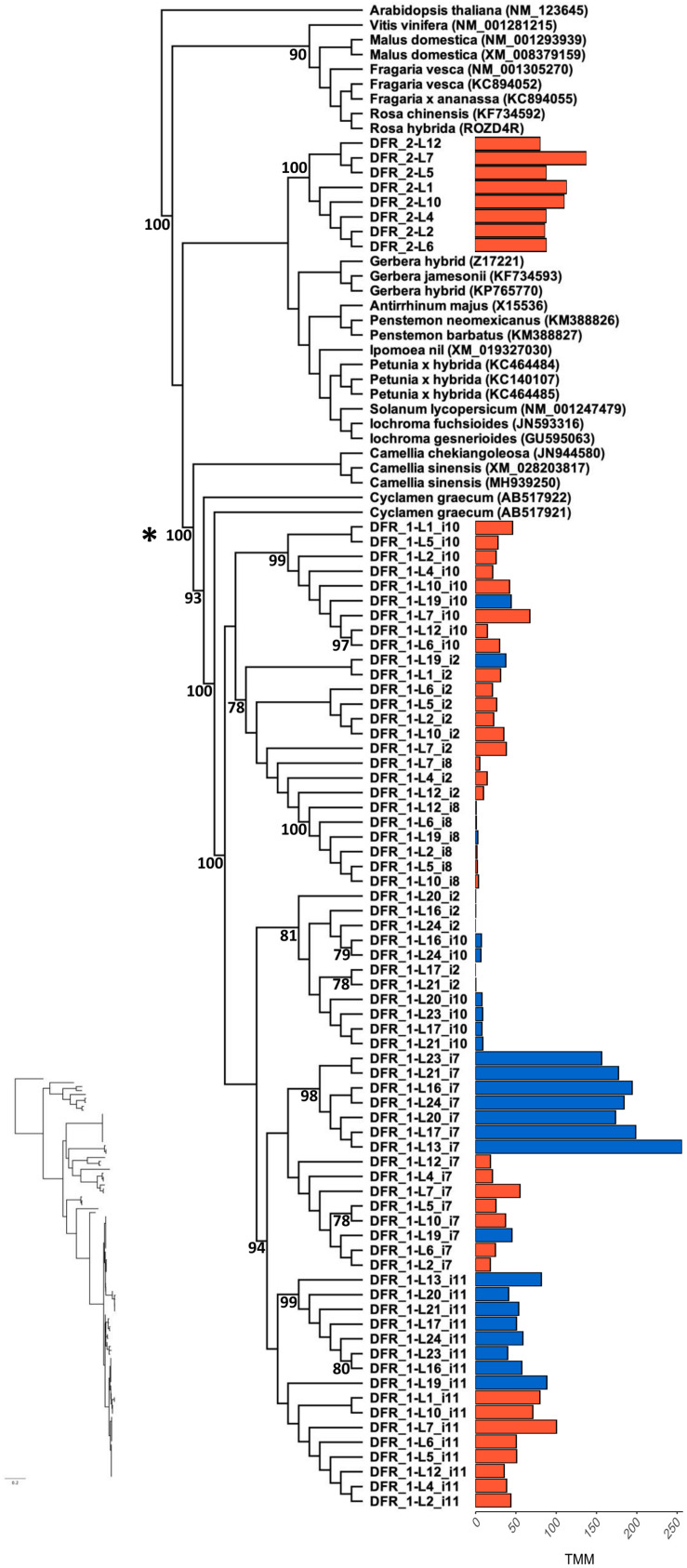
Maximum likelihood phylogenetic analysis of *DFR-1* and *DFR-2* coding sequence for all *L. arvensis* isoforms (L1–12 = orange; L13–24 = blue). We included the top BLASTn hits for reference (scientific name followed by followed by Genbank Accession numbers). Bootstrap values provided along the branch or at the node for values above 70%. We removed he 5′ and 3′ ends (24 and 52 bp, respectively) of the coding sequence because of alignments ambiguities. The bar plot shows the expression value (TMM) per isotig for blue and orange flower colors. The isotigs with markedly shorter coding sequences than the Genbank references are *DFR-1-i7*, *DFR-1-i8*, and *DFR-1-i11*. *Indicates the location of the putative gene duplication event. The maximum likelihood phylogram without tip labels (yet same order) is included to allow for relative branchlength comparisons.

The phylogenetic analyses of the two forms of *DFR* indicate that there are two distinct lineages of *DFR* (*DFR-1* and *DFR-2*) and that *L. arvensis* samples of each are both reciprocally monophyletic yet the two *DFR*s are deeply diverged within angiosperms (i.e., *DFR-1* and *DFR-2* diverged long before the origin of *Lysimachia*) (Figure 4 and Supplementary Figure 4). The clade of orange-flowered *DFR-2* samples is very strongly supported (100% bootstrap support). It is sister to several Genbank reference samples from the Asterids (members of the Asteraceae, Solanaceae, Convolvulaceae, Plantaginaceae). Alternatively, *DFR-1* forms a very strongly supported clade (100% bootstrap) that is closely related to two samples from the Ericales (*Cyclamen graecum* and *Camellia spp.*). There is very strong support for a clade containing *DFR-1 Camellia* and *Cyclamen* to the exclusion of *DFR-2* and the Asterid samples (100% bootstrap). There are 74 non-synonymous amino acid changes separating *DFR-1* and *DFR-2*. Five of these are variable in *DFR-1*, but fixed in *DFR-2* and one is variable in *DFR-2*, but fixed in *DFR-1*. This includes five amino acid replacements in known functional domains (see “SNP frequencies” subsection below). Since there were no blue *DFR-2* sequences created during the reference-guided assembly, all further analyses comparing orange- and blue-flowered samples focus on *DFR-1*.

For the *DFR-1* clade, there are two patterns. The first pattern clusters isotigs into monophyletic clades with other isotigs of the same color (e.g., orange-i10, i2 and i8 form a strongly supported clade and blue-i10 and blue-i2 form a moderately supported clade; Figure 4). The second pattern consists of blue samples of one isotig which are sister to the orange samples from the same isotig (e.g., i7 and i11). Similar to the results for *F3′5′H*, the blue sample L19 falls within orange clades for all isotigs, sometimes with very strong bootstrap support, consistent with it being a hybrid.

### SNP Frequencies for *F3′5′H* and *DFR*

**FIGURE 5:**
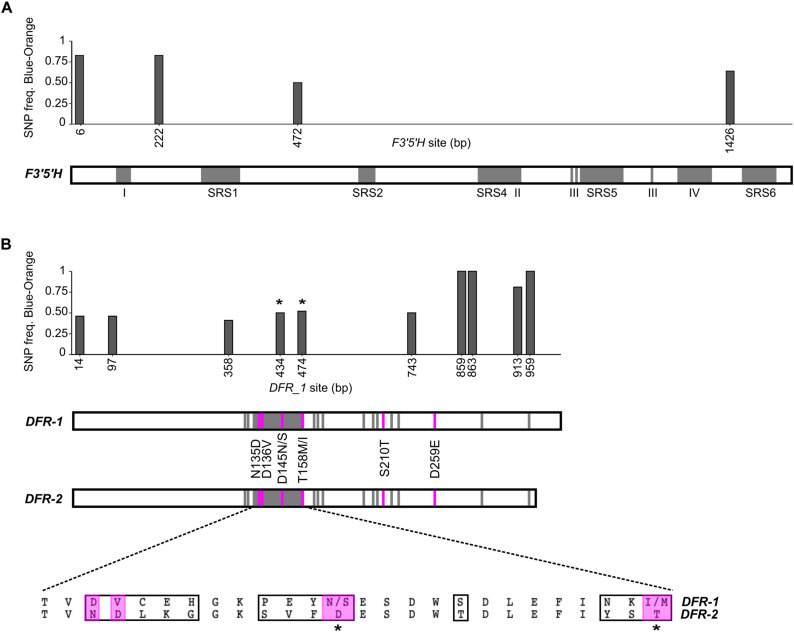
Non-synonymous SNP analysis of *F3′5′H* and DFR for *L. arvensis*. SNP frequency graphs are aligned with the gene diagram indicating known functional domains (gray). For *F3′5′H* **(A)** there are five binding regions (SRS; Seitz et al., 2007) and other known functional domains (I-IV; Guo et al., 2019). The DFR analysis **(B)** shows non-synonymous SNP frequencies in DFR-1 in the graph. Gene diagrams show the substrate specificity region (amino acid sites 133–158; Johnson et al., 2001) and other previously identified substrate binding sites (Petit et al., 2007; Smith et al., 2012) in gray. Amino acid changes between blue and orange DFR-1 located in a substrate binding region are marked with *. Amino acid changes between DFR-1 and DFR-2 identified in other works to be relevant to the substrate specificity differentiation between colors (Petit et al., 2007; Smith et al., 2012) are indicated in the diagram (pink) and their location and single letter abbreviation are shown between the DFR diagrams. An alignment of DFR-1 and DFR-2 consensus sequences of the 26 amino acids from the substrate specificity region is provided, highlighting all the amino acid changes in a black box (sites 133-158; relevant amino acid changes shown in the gene diagram are highlighted in pink).

We investigated SNP frequencies that correlate with flower color in two candidates within the narrowly defined ABP genes. For *F3′5′H*, there were four non-synonymous SNPs in the CDS (Figure 5A). Two of these were fixed for all orange samples in all isotigs and variable in all blue samples for all isotigs (bp472 and bp1426; Table 2). Both had BLOSUM62 scores of +1 (meaning relatively common amino acid replacements), yet each of them involved significant changes in the biochemistry of the amino acids involved (e.g., the SNP at bp472 changes from a positively charged H to a polar uncharged N and the SNP at bp1426 changes from a positively charged K to a negatively charged E). There were 18 additional synonymous SNPs in the *F3′5′H* CDS most of which were variable in blue samples, but not in orange samples (Supplementary Table 7). Over all 22 *F3′5′H* SNPs (synonymous and non-synonymous), the frequency differences between blue- and orange-flowered samples (across isotigs) ranged from 0.09 to 0.83 (mean 0.53). There were an additional five SNPs in the 5′-UTR and six SNPs in the 3′-UTR. The average difference between blue- and orange-flowered SNP frequencies in the UTRs was 0.57 (0.09–0.79). None of the non-synonymous SNPs for *F3′5′H* were located in any of the known functional domains (Figure 5A).

**TABLE 2:** Comparison of amino acid (AA) variation in the *F3′5′H* non-synonymous SNP sites of the coding sequences between blue and orange *L. arvensis* isotigs.

	Isotig	Site 6	Site 222	Site 472	Site 1426
Blue^∗^	i1	E	K	N/H	K/E
	i2	E	K	N/H	K/E
	i3	E	K	N/H	K/E
	i4	E	K	N/H	K/E
	i9	E	K	N/H	K/E
	i10	E	K	N/H	K/E
Orange	i1	E	K	N	E
	i2	E	K	N	E
	i3	E	K	N	E
	i4	D	N	N	E
	i9	E	K	N	E
	i10	E	K	N	E
BLOSUM62 score		+2	0	+1	+1

Biochemistry		Both negatively charged	Positive (K) to polar uncharged (N)	Positive (H) to polar uncharged (N)	Positive (K) to negative (E)

Site numbers refer to base pairs (bp). *Not including blue sample L19, which is closely related to orange samples.

Since there are well-characterized functional domains in *DFR* that provide substrate specificity conferring blue-to-red shifts in other species (Johnson et al., 2001; Petit et al., 2007; Des Marais and Rausher, 2008; Smith et al., 2012), we compared *DFR-1* and *DFR-2* for fixed amino acid differences at these sites. For the 26 residue substrate specificity region identified by Johnson et al. (2001) (amino acids 133 to 158 in our alignment), the *L. arvensis DFR-2* has fixed differences in 13 of them compared to *DFR-1* (Figure 5B). For the additional 12 substrate binding sites identified by Petit et al. (2007), we found only one site (amino acid 210) where *DFR-2* was fixed with a serine (S) compared to a threonine (T) in *DFR-1* (BLOSUM62 score = +1; both polar uncharged amino acids). Two more color-differentiating sites identified by Smith et al. (2012) were fixed in both *DFR*s (Figure 5B). In addition to these five amino acid substitutions in known functional domains, we identified 63 more amino acid changes outside known functional domains that were fixed between *DFR-1* and *DFR-2* and another six that were variable in one copy of *DFR*, yet fixed in the other.

Since *DFR-2* is only expressed at considerable levels in orange flowers, we focus our color-differentiating SNP analysis on *DFR-1* where we have sequences of both blue- and orange-flowered individuals. Over all 54 *DFR-1* SNPs (synonymous and non-synonymous), the frequency differences between blue- and orange-flowered samples (across isotigs) ranged from 0.12 to 1 (mean 0.46). There were ten non-synonymous SNPs with considerable differences in frequency between flower colors (Table 3). The non-synonymous SNPs located in the binding specificity region (bp434 and bp474; Figure 5B) showed clear patterns of color frequency differences between the two flower color types (bp434 = 0.50 SNP frequency difference; bp474 = 0.52 SNP frequency difference; Supplementary Table 8). Both have a BLOSUM62 score of +1 and are amino acid substitutions within the same general biochemical category (bp434 = both polar uncharged amino acids; bp474 = both hydrophobic amino acids). Three non-synonymous SNPs in the 3′ half of the *DFR-1* coding sequence that (only present in i2 and i10) were completely differentiated between blue- and orange-flowered samples (bp859, bp863, and bp959). Their BLOSUM62 scores range from 0 to +2 and two of them represent biochemically distinct amino acid replacements (bp859 and bp913 = negatively charged to polar uncharged amino acids; Table 3). There were 44 additional synonymous SNPs in the *DFR-1* CDS (Supplementary Table 8).

**TABLE 3:** Comparison of amino acid (AA) variation in the *DFR_1* non-synonymous SNP sites of the coding sequences between blue and orange *L. arvensis* isotigs.

	Isotig	Site 14	Site 97	Site 358	Site 434	Site 474	Site 743	Site 859	Site 863	Site 913	Site 959
Blue*	i10	I/T	T	K/G	N/S	I/M	E	E	S	E	N
	i11	T	T	E	N	I	–	–	–	–	–
	i2	I	T	K/G	N/S	I/M	E	E	S	E	N
	i7	I	T	T	N	M	–	–	–	–	–
Orange	i10	T	T	K	S	I	A	Q	N	Q	T
	i11	T	T	K	N	I	–	–	–	–	–
	i2	I	S/T	K	S	I	E	Q	N	Q/E	T
	i7	I	S/T	T/I	N	I	–	–	–	–	–
	i8	I	S/T	K	S	I	–	–	–	–	–
BLOSUM62 score		−1	+1	NA	+1	+1	−1	+2	+1	+2	0

Biochemistry		Hydrophobic (I) to polar uncharged (T)	Both polar uncharged	NA	Both polar uncharged	Both hydrophoabic	Hydrophobic (A) to negative charged (E)	Negatively charged (E) to polar uncharged (Q)	Both polar uncharged	Negatively charged (E) to polar uncharged (Q)	Both polar uncharged

Site numbers refer to base pairs (bp). *Not including blue sample L19, which is closely related to orange samples.

### Identification of Flavonoid Compounds by UHPLC-MS

**FIGURE 6:**
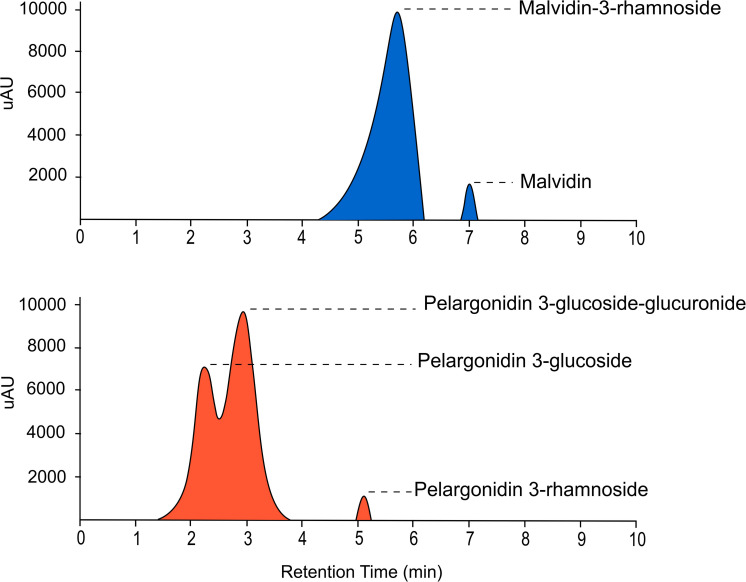
UHPLC-MS chromatograms of the main anthocyanins present in blue (above) and orange (below) petals of *L. arvensis* detected at 520 nm.

**FIGURE 7:**
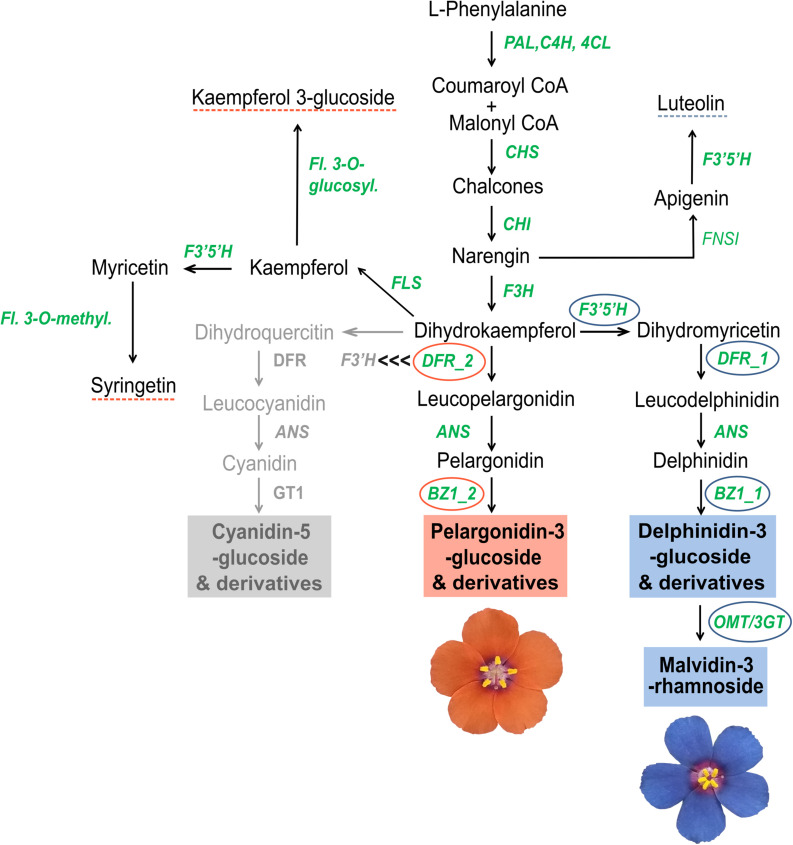
Tentative scheme of the anthocyanin biosynthetic pathway for *L. arvensis* petals. Structural genes involved in the broader flavonoid biosynthetic pathway are shown in green: *Phenylalanine ammonia-lyase (PAL*), *Cinnamic 4-hydroxylase* (*C4H*), *4-coumarate-CoA ligase* (*4CL*), *Chalcone synthase* (*CHS*), *Chalcone isomerase* (*CHI*), *Flavanone 3-hydroxylase* (*F3H*), *Flavonoid 3′*,*5′-hydroxylase* (*F3′5′H*), *Dihydroflavonol 4-reductase* (*DFR*), *Anthocyanin synthase* (*ANS*), *Anthocyanidin-3-o-glucosyltransferase* (*BZ1*), *Anthocyanin-3′-o-beta-glucosyltransferase* (*3GT*), *O-methyltransferase* (*OMT*), *Flavonol synthase* (*FLS*), *Flavonol-3-o-glucosyltransferase* (*Fl. 3-o-glucosyl.*), *Flavone synthase I* (*FNSI*), and *Flavonol 3-O-methyltransferase* (*Fl. 3-O-methyl.*). Narrow ABP genes differentially expressed in orange and blue *L. arvensis* are marked with orange and blue circles, respectively. Blue and orange boxes mark the anthocyanin compounds found by UHPLC-MS in each flower color. Blue and orange dashed lines mark the non-anthocyanin compounds found by UHPLC-MS in each flower color. The gray portion indicates the location of the inactivated branch leading to cyanidin-derivatives that would exist if *F3*′*H* outcompeted *DFR-2* for dihydrokaempferol. We hypothesize that *DFR-2* outcompetes *F3*′*H* at this step suggested by the black “<<<” symbols.

Ultra-High Performance Liquid Chromatography analysis confirmed that malvidin-3-rhamnoside is the main flavonoid that accumulates in the blue petals of *L. arvensis* (relative content = 98.4%; Table 4 and Figure 6). In addition, blue petals also contain traces of the aglycone malvidin and delphinidin derivatives. In contrast, orange petals contain four derivatives of pelargonidin with different glycosylation patterns: pelargonidin-3-glucoside (60.1% relative content), pelargonidin-3-glucoside-glucuronide (26.5%), pelargonidin-3-rhamnoside (11.6%), and pelargonidin-3-diglucoside (trace amounts; Table 4 and Figure 6). We also detected traces of the flavone luteolin-7-glucoside in the blue samples, and small amounts of two flavonols present in some orange samples – syringetin (trace) and kaempferol-3-glucoside (1.7%). The putative locations of these compounds in the flavonoid biosynthetic pathway are shown in Figure 7.

**TABLE 4:** Putative flavonoid identification in blue and orange petals of *L. arvensis* from the UHPLC–MS biochemical analysis (MS analysis was acquired in positive mode).

Petal color	Flavonoid type	Putative flavonoid identification	N samples*	*t*_*R*_ (min)	*λ_*max*_* (nm)	Mass (m/z)	Relative content (%)**
**Blue**	Anthocyanin	Delphinidin 3-glucoside-rhamnoside	1/14	2.3	233, 302, 522	609.90	trace
(14 plants)	Anthocyanin	Delphinidin 3-rhamnoside-rhamnoside	1/14	3.3	235, 310, 522	592.45	trace
	Anthocyanin	Malvidin 3-rhamnoside	14/14	5.8	227, 280, 527	476.71	98.4
	Anthocyanin	Malvidin	1/14	7.0	231, 268, 350, 533	331.28	1.6
	Flavone	Luteolin 7-glucoside	2/14	7.9	230, 290, 340, 377	448.48	trace
**Orange**	Anthocyanin	Pelargonidin 3-glucoside-glucuronide	4/11	2.2	279, 427, 502	606.66	26.5
(11 plants)	Anthocyanin	Pelargonidin 3-diglucoside	2/11	2.8	239, 280, 428, 500	594.72	trace
	Anthocyanin	Pelargonidin 3-glucoside	11/11	3.0	283, 427, 502	433.09	60.1
	Anthocyanin	Pelargonidin 3-rhamnoside	6/11	5.2	232, 268, 483, 506	416.72	11.6
	Flavonol	Syringetin	3/11	6.9	232, 264, 294, 346	346.64	trace
	Flavonol	Kaempferol 3-glucoside	5/11	7.9	232, 264, 294, 346	448.75	1.7

Petals do not include the darker basal area (i.e., bullseye). Identification was based on retention time (t_R_), maximum absorbance wavelength (λmax) observed in the UV-Vis spectra of the compound that eluted at the t_R_ indicated, and molecular mass comparisons with standards and values of previously reported flavonoids for Lysimachia spp. (see Supplementary Table 2 and the database http://metabolomics.jp). *The number of samples where each flavonoid was observed. **Relative content of each putative compound in each flower color, calculated from the areas of the UV-Vis peaks.

## Discussion

We found 38 genes with significant differential expression between flower colors of *L. arvensis* across 94 genes in the flavonoid biosynthetic pathway. Our two sampled populations are geographically separated and may represent incipient species based on recent phylogenetic results (Jiménez-López, 2019; Jiménez-López et al., in review), which may have introduced some differences between the populations not correlated with color. Nevertheless, we still detected patterns correlated with color that are consistent with known functions of the ABP loci and previous studies of comparable flower color transitions. First, we focus on the ABP genes including discussion about the expression differences, SNP detection and biochemical changes associated with two enzymes at a critical branchpoint in the ABP in order to shed light on the molecular underpinnings of the blue to orange shift in *L. arvensis*. Then, we expand the conversation to include additional genes in the flavonoid biosynthetic pathway not previously associated with such a color shift and discuss potential pleiotropic consequences not directly related to pollinator attraction. Finally, we mount evidence for a directional transition from blue to orange flowers in *L. arvensis*.

Within the narrow ABP, there were two DEGs in *L. arvensis* concentrated in the latter half of the pathway that are likely directly involved in the blue to orange shift in flower color (*F3′5′H* and *DFR*). These genes had the highest overall expression of all the narrowly defined ABP genes. This flower color transition could be caused by either of these or some combination of the two since these genes are responsible for the synthesis of two different types of anthocyanins: delphinidin-derivatives (in the case of *L. arvensis* this is malvidin that produces the blue color) and pelargonidin-derivatives (orange; Figure 7; Smith and Rausher, 2011; Smith et al., 2012; Wheeler and Smith, 2019). These biochemical results are consistent with previous findings in *L. arvensis* (Lawrence et al., 1939; Harborne, 1968; Ishikura, 1981; Kawashty et al., 1998). Several additional ABP genes with differential expression in blue- vs. orange-flowered plants were consistent with the post-translational modifications of these principle pigments. Here, we discuss three of these genes (Figure 2). *BZ1-2* showed high levels of expression in orange petals (TMM > 290) compared to blue (expression 950× O > B). This enzyme introduces a glucose group to the 3-position of the anthocyanin precursor’s C-ring to stabilize the color (Koes et al., 2005). The high expression of *BZ1-2* in orange-flowered *L. arvensis* plants correlates with the biochemical results showing that orange petals are composed of pelargonidin 3-glucoside derivatives whereas blue petals are malvidin glycosylated with rhamnoside. Similarly, enzymes *OMT* and *3GT* showed higher levels of expression in blue than orange samples (5.3× and 2.9×, respectively). *OMT* methylates delphinidin to form malvidin, and *3GT* introduces the rhamnoside (Saito et al., 2013) both of which are unique to blue flowered samples, likely involved in stabilization and protection of the pigment, but unlikely solely responsible for the color change.

### Role of *F3′5′H* Expression and Amino Acid Substitutions

*F3′5′H* catalyzes the hydroxylation of dihydrokaempferol to produce a delphinidin precursor (Halbwirth, 2010). Our expression results indicate a 2.5× decrease in expression in orange petals consistent with several previous studies investigating the blue- to orange-flowered shift among a diversity of eudicots (Rausher, 2008; Wessinger and Rausher, 2014; Larter et al., 2018). For example, the loss of function of *F3′5′H* in *Iochroma* spp. (Smith and Rausher, 2011), *Antirrhinum* spp. (Ishiguro et al., 2012) and *Penstemon barbatus* (Wessinger and Rausher, 2013) or the decreased expression of *F3′5′H* in *Phlox drummondii* (Hopkins and Rausher, 2011), confers blue to red color shifts. Rausher (2008) suggests that unidirectional changes at this key step in the ABP play an important role in the repeated transitions from blue to red/orange flowers observed in many angiosperm lineages. In *L. arvensis*, *F3′5′H* activity is not completely lost since *F3′5′H* isotigs were detected (at low levels) in orange petals and we detected traces of a flavonol requiring *F3′5′H* activity (syringetin) in almost 30% of the orange petal samples. In addition to the decreased expression of *F3′5′H*, there are four flower color differentiating non-synonymous SNPs that may contribute to the blue to orange shift, but would require future enzyme activity assays to be determined.

### Role of *DFR* in the Blue to Orange Transition

The transition from delphinidin-like precursors (malvidin in this case) to pelargonidin precursors caused by changes in *F3′5′H*, creates a new substrate which requires subsequent modifications in downstream enzymes like *DFR*. Several lines of evidence suggest that *DFR* in *L. arvensis*, which catalyzes the reduction of dihydroflavonols to leucoanthocyanins, may also be involved in the evolutionary transition from blue to orange flowers. Our transcriptome assembly identified two distinct copies of *DFR*. One copy, *DFR-2*, is expressed primarily in orange flowered individuals (nearly undetectable in blue samples). This copy presents an N residue in the third position of the substrate specificity region (residue 135 in *L. arvensis*), which is known to confer substrate affinity to dihydrokaempferol, the pelargonidin-like precursor, in a wide group of angiosperms (Johnson et al., 2001; Shimada et al., 2005). Alternatively, *DFR-1* is primarily expressed in blue-flowered individuals and exhibits a D residue at this site, which confers less or no substrate affinity for dihydrokaempferol in other species (Johnson et al., 2001; Shimada et al., 2005). We propose that *DFR-1* in *L. arvensis* is likely more efficient in processing dihydromyricetin, the delphinidin-like precursors to malvidin. Like *F3′5′H*, a combination of expression and changes in the amino acid composition of *DFR* likely contributes to the completion of the blue to orange color transition. In *Petunia*, alteration of the amino acids in the substrate specificity domain of *DFR* confers a flower color shift by increasing catalytic efficiency in metabolizing dihydrokaempferol, a pelargonidin precursor (Johnson et al., 2001). In addition, three more amino acid changes between *DFR-1* and *DFR-2* have been suggested to be involved in substrate specificity differentiation in other species. In *Iochroma* and *Vitis* (Petit et al., 2007; Smith et al., 2012), the number of sites responsible for substrate specificity is larger and more physically spread out along the *DFR* coding region than the 26 amino acid stretch suggested by Johnson et al. (2001). All these amino acid changes between the two copies of *DFR* present in *L. arvensis* are correlated with blue to orange flower color transition, yet causality would require further genetic manipulation.

Within the *DFR-1* coding sequence of *L. arvensis*, there are many color differentiating SNPs. There are two amino acid changes nearly fixed between colors that also differ from *DFR-2*, and are located in the 26 amino acid substrate specificity region, suggesting interesting residue positions for future studies of differential substrate binding affinity. Overall, we suggest that *DFR-1* may have had an original affinity for dihydromyricetin, which is maintained in blue flowers, while in orange-flowered individuals, a trans-regulatory change to *DFR-2* expression (e.g., via *LaMYB61* O > B) may confer dihydrokaempferol specificity to accommodate the new substrate being produced by the loss of *F3′5′H* activity. If *DFR-2* outcompetes *F3*′ for dihydrokaempferol as described in other angiosperms producing pelargonidin (Des Marais and Rausher, 2008), then the flux would be directed to the pelargonidin pathway (even if *F3*′ enzymes are present as we have relatively equal expression in blue and orange-flowered samples for *F3*′). In order to produce orange flowers, there must be no (or very low) expression of *DFR-2* in blue *L. arvensis* to allow *F3′5′H* to win the competition for dihydrokaempferol redirecting the flux down the delphinidin branch of the ABP.

The maintenance and detectable expression of both *DFR-1* and *DFR-2* in *L. arvensis* is somewhat enigmatic. A similar situation exists in the Convolvulaceae with three *DFR* copies of relatively recent origin in that family (Des Marais and Rausher, 2008). However, the duplication of *DFR-1* and *DFR-2* we report is likely independent of the gene duplications reported by Des Marais and Rausher (2008), because it appears to be much older, perhaps predating the split of Asterales and Ericales (see annotation in Figure 4). Next, we plan to investigate the petal transcriptomes of the closely related *L. monelli*, which exhibits phenotypically similar petal color polymorphism. We expect to find two copies of *DFR* in that case, as well, and will compare its color-specific expression differences to those we report for *L. arvensis* herein.

### Broader Insights From the Flavonoid Biosynthetic Pathway and the Roles of Non-pollinator Agents of Selection

We hypothesized that there would be some flower-color specific differences in the transcriptome for genes outside the narrow ABP that might open the door for agents of selection not associated with pollinator attraction (Strauss and Whittall, 2006; Rausher, 2008; Ortiz-Barrientos, 2013). In this regard, we found 30 DEGs in the broader flavonoid pathway, outside the narrow ABP (15 DEGs with B > O with an average fold-change of 9.1 and 15 DEGs with O > B with an average fold-change of 18.8). Among these non-ABP DEGs, we looked for loci with potential functionality that may correlate with the ecological differences of the two color types (Arista et al., 2013). For example, *Caffeoyl CoA O-methyl* (only present in blue samples with TMM = 1.27) and *Caffeoyl 3-O-methyl* (*ATTSM1-1* and *ATTSM1-2*, with B > O and an average fold-change of 4.8 and 3.7, respectively) both promote anthocyanin methylation following drought stress in *Vitis vinifera* (Giordano et al., 2016) and have been implicated in lignin biosynthesis, an important barrier against pathogens (Liu et al., 2018). Additionally, we found differential expression for three *CYP84A* genes with O > B fold-changes ranging from 3.3 to 28.1. These loci are involved in sinapate ester biosynthesis, a key flavonoid compound providing UV protection (Ruegger et al., 1999). Finally, in defense against herbivores and pathogens, we found differential expression of two *IEMT* (with O > B average fold-change of 3.3 and 186.8) involved in the synthesis of isomethyleugenol and methyleugenol for deterring herbivores (and/or attracting pollinators; Barkman, 2003) and two *PKR* (also known as *CHR;* one with B > O fold-change of 30.4, and the other with O > B fold-change of 2.3) which produces phytoalexins (isoflavonoids, coumestans, pterocarpans, and isoflavans) in response to herbivore or pathogen attack in legumes (Bomati et al., 2005).

The most dramatic expression differences we found between blue and orange *L. arvensis* petal transcriptomes are those in the ABP genes. The average fold-change of all significantly differentially expressed ABP genes is 204×, but only 14× for non-ABP flavonoid pathway genes. Furthermore, most ABP overall expression levels are much higher than all other flavonoid pathway genes combined. The relative importance of the ABP vs. non-ABP genes are consistent when correcting for color-type (orange ABP gene expression is 4.8× higher than orange non-ABP gene expression; Wilcoxon rank-sum test, *p* = 1.3 × 10^–12^; blue ABP gene expression is 6× higher than blue non-ABP gene expression; Wilcoxon rank-sum test, *p* = 8.5 × 10^–9^). A notable exception to the low expression of non-ABP flavonoid pathway genes is flavonol synthase (FLS). This side-branch in the ABP catalyzes the synthesis of flavonols from dihydroflavonols. In *L. arvensis* petals, *FLS* has very high expression in blue samples (TMM > 500) and has 3.5× higher expression in blue- vs. orange-flowered samples (Figure 2 and Table 1). In *Arabidopsis, FLS* has the highest expression in petals, and it can compete with *DFR* for flux down the anthocyanin portion of the ABP (Nguyen et al., 2016; McCarthy et al., 2017). It is often involved in plants’ abiotic stress response (Lee et al., 2016) neutralizing damaging reactive oxygen species (Li B. et al., 2019). In contrast, activation or inactivation of *FLS* is involved in pollinator shifts in *Petunia* species (Esfeld et al., 2018). Nevertheless, our UHPLC results lack any measurable levels of flavonols (over 98% of detected flavonoids were anthocyanins in both color types; Table 4). This suggests that either (a) *DFR* is such a good substrate competitor that, even with high expression of *FLS*, the flux continues toward anthocyanin production, or (b) the main activity of *FLS* is not for flavonol production, but may be involved in some other undescribed pathway.

The transcriptome results for the broader flavonoid biosynthetic pathway genes are largely confirmed by our biochemical survey. Although the predominant flavonoids extracted and detected in *L. arvensis* petals were blue and orange anthocyanins, we also found three additional flavonoids representing intermediates and side-branches to the ABP. The flavone luteolin 7-glucoside was previously found in blue petals of *L. arvensis* (Ishikura, 1981) and two flavonols were present in our orange samples: kaempferol 3-glucoside (also identified in Kawashty et al., 1998) and syringetin (this is the first record for compound in *L. arvensis*, although it was previously reported in *L. congestiflora* according to Guo et al., 1998). These compounds are known to have some functions outside of traditional pollinator attraction (Winkel-Shirley, 2002; Jiang et al., 2016). Despite their detection in our UHPLC analysis, they were rare (kaempferol = 1.7% of total flavonoids extracted and the other two were only detected in trace amounts). Beyond these steps, which are very close to the narrow ABP, our biochemical extraction technique and flavonoid references employed may have limited our ability to detect other flavonoid compounds that may respond to non-pollinator agents of selection.

Our results suggest that flavonoid biosynthetic pathway genes may play additional roles beyond pollinator attraction. The transcriptome and biochemical results clearly indicate that the vast majority of ABP enzymes and biochemicals in petals of *L. arvensis* are primarily focused on anthocyanin production. Whether the anthocyanin compounds in the petals have functions beyond pollinator attraction could not be directly discerned in this study. Previous work by Arista et al. (2013) found that natural populations of blue-flowered *L. arvensis* were more commonly found in the hotter and drier environments of southern Europe in contrast to orange-flowered populations that predominate in the north. Further, their manipulative experiments showed higher fitness for blue-flowered individuals when faced with water stress (Arista et al., 2013). *Lysimachia arvensis* has distinctive cyanidin pigment spots on the undersides of the leaves with unknown function (unpublished data). The predominance of cyanidin anthocyanins in angiosperm vegetative tissues (and lack of pelargonidin-based anthocyanins) regardless of flower color suggests an evolutionary constraint that filters for only tissue-specific changes in floral anthocyanins (Price and Sturgess, 1938; Streisfeld and Rausher, 2011). This correlates with known differences in antioxidant function between malvidin and pelargonidin (blue and orange petals, respectively). The most important determinant of antioxidant activity for anthocyanins is the hydroxylation level of the B-ring (Halbwirth, 2010). Malvidin has three hydroxyl groups whereas pelargonidin has only one and malvidin has much higher antioxidant activity than pelargonidin (Kähkönen and Heinonen, 2003). This may explain why blue-flowered individuals have higher fitness in xeric and high temperatures compared to orange-flowered individuals. Flower life-span is also longer in blue flowers, as every day they open earlier and close latter than orange flowers (Jiménez-López, 2019). Whether this is due to the different anthocyanins in the petals or pleiotropic effects in vegetative tissues is still unknown.

### Directional Change in Color From Blue to Orange

The direction of the flower color change in *L. arvensis* remains uncertain although there are clear predictions (Rausher, 2008) and our findings are consistent with these predictions. The directional change from blue to red (and rarely the reverse) is often attributed to the loss of function at a key branchpoint in the ABP (*F3*′ and *F3′5′H*) responsible for the blue/purple pigment formation (delphinidin and cyanidin). Without these branches, the precursor, dihydrokaempferol, is shunted directly down the red pathway (pelargonidin; Zufall and Rausher, 2004; Rausher, 2008; Smith and Rausher, 2011). We find a 2.5× decrease in *F3′5′H* expression in orange- compared to blue-flowered samples (coupled with color-differentiating non-synonymous SNPs). In a recent review of the genetic bases for flower color transitions, *F3′5′H* or/and *F3*′ were implicated in all 10 blue to red transitions documented from natural systems (see Supplementary Table 3 in Wheeler and Smith, 2019). Following the inactivation of the delphinidin branch of the ABP and the redirection of flux down the pelargonidin branch, subsequent evolution of substrate specificity by recruiting *DFR-2* (expression ∼600× O > B) instead of the delphinidin-targeted *DFR-1*, would compete for any remaining *F3*′ expression to metabolize dihydrokaempferol down the pelargonidin branch (Johnson et al., 2001; Zufall and Rausher, 2004; Smith and Rausher, 2011). Wheeler and Smith (2019) find only two of ten examples from blue to red transitions implicated changes in *DFR*, suggesting that this may be a secondary modification after the initial mutation of large effect (decreased activity of *F3′5′H*). Finally, subsequent changes, such as increased substrate specificity for *BZ1* and other post-translational modifications (e.g., glycosylation) would eventually produce pelargonidin-3-glucoside (Figure 7). By coupling tissue-specific expression changes in *F3′5′H* and transitioning from *DFR-1* to *DFR-2*, these evolutionary changes may arrive with very little pleiotropic cost to the organism (Streisfeld and Rausher, 2011), especially if expression changes are restricted to petals via trans-regulatory changes (e.g., *LaMYB61*).

In addition to the predictions based on gene expression and substrate specificity, we found additional evidence consistent with a blue-to-orange transition when examining the *F3′5′H*. In the SNP survey, blue samples were often variable (18/22 = ∼81%) whereas in orange samples, only five SNPs were variable (5/22 = 23%). This pattern is consistent with a selective sweep during the transition from blue to orange causing a reduction in variation in the orange samples (although there are other demographic patterns that could be responsible). Further evidence for a blue-to-orange transition in *L. arvensis* comes from the *Lysimachia* ITS phylogeny indicating that blue is the ancestral flower color for *L. arvensis* and the orange-flowered individuals are the derived state (Jiménez-López, 2019; Jiménez-López et al., in review).

## Conclusion

The transcriptomic and biochemical analysis of the flavonoid biosynthetic pathway presented in this study provides an important foundation for understanding the molecular genetic basis of flower color polymorphism in *L. arvensis*. The differential expression between flower colors, particularly along the ABP branch, is consistent with the main anthocyanin compounds found in blue and orange petals. As we hypothesized, *F3′5′H* and *DFR* are the principal genes guiding dihydrokaempferol to the proper anthocyanin formation. Our study provides a valuable resource for future molecular studies of *Lysimachia* flowers and sheds light on more general evolutionary processes underlying the diversity of flower color variation.

## Data Availability Statement

All raw sequence to the NCBI SRA database under BioProject accession number PRJNA701383. Phylogenetic analyses of F3’5’H and DFR have been placed on Figshare at doi: 10.6084/m9.figshare.13911287.v1.

## Author Contributions

MA, PO, EN, JW, and FJ-L conceived and designed the experiments. JW and FJ-L carried out the sampling. FR-C, KR, and BI performed the assembly. KR, BI, and MS-C performed the differential expression analysis. AF developed the UHPLC-MS methods. EN and MS-C analyzed the UHPLC-MS data. MA, PO, EN, JW, FJ-L, and MS-C drafted the manuscript. All authors read and approved the final manuscript.

## Conflict of Interest

The authors declare that the research was conducted in the absence of any commercial or financial relationships that could be construed as a potential conflict of interest.

## Footnotes

1 https://www.genome.jp/kegg/pathway.html

2 https://github.com/karolisr/kakapo

3 https://blast.ncbi.nlm.nih.gov/Blast.cgi

4 http://metabolomics.jp/wiki/Index:FL
