# Supplementary material for: Changes at a Critical Branchpoint in the Anthocyanin Biosynthetic Pathway Underlie the Blue to Orange Flower Color Transition in Lysimachia arvensis
Source: Front Plant Sci. 2021 Feb 22;12:633979. doi: 10.3389/fpls.2021.633979 (PMC7937975; doi:10.3389/fpls.2021.633979)
Supplement: Supplementary file 1 [file Data_Sheet_1.zip › Supplementary Figures.pdf]

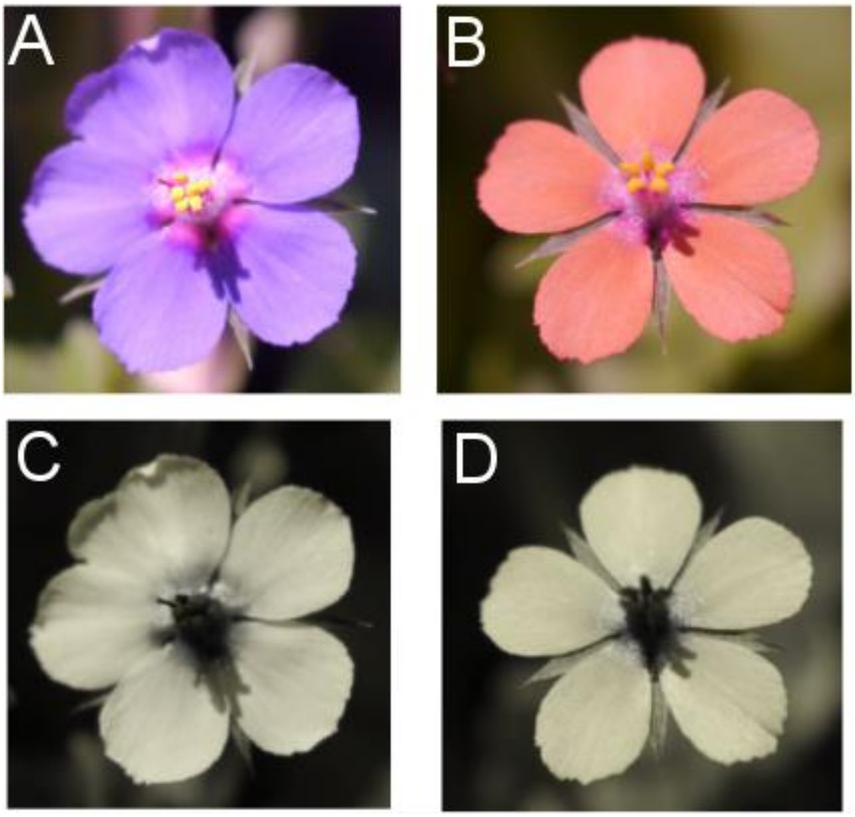

Supplementary Figure 1. Digital photos of blue and orange *L. arvensis* flowers documented with visible light (A & B) and UV light (C & D) showing no dramatic difference in the bullseye. Plants were grown in a greenhouse from seed collected from natural populations from Mediterranean Europe.

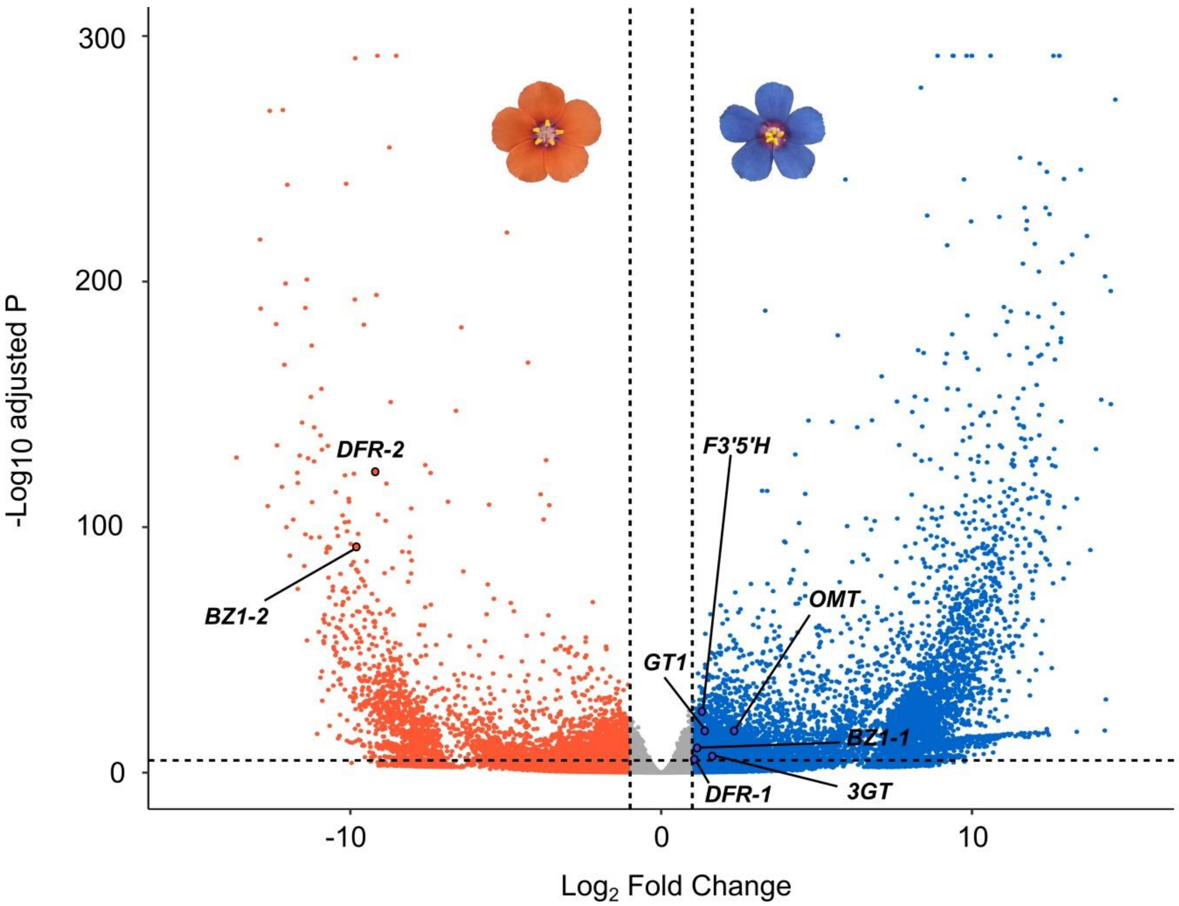

Supplementary Figure 2. Volcano plot of differentially expressed genes (DEGs) between blue and orange petals of *L. arvensis*. Orange dots represent genes with higher expression in orange petals and blue dots represent genes with higher expression in blue petals. Dashed lines mark the threshold for significant DEGs: -log10 adjusted P < 10-5 and log2FC >1. In black are highlighted the structural DEGs of *L. arvensis* from the narrow ABP.

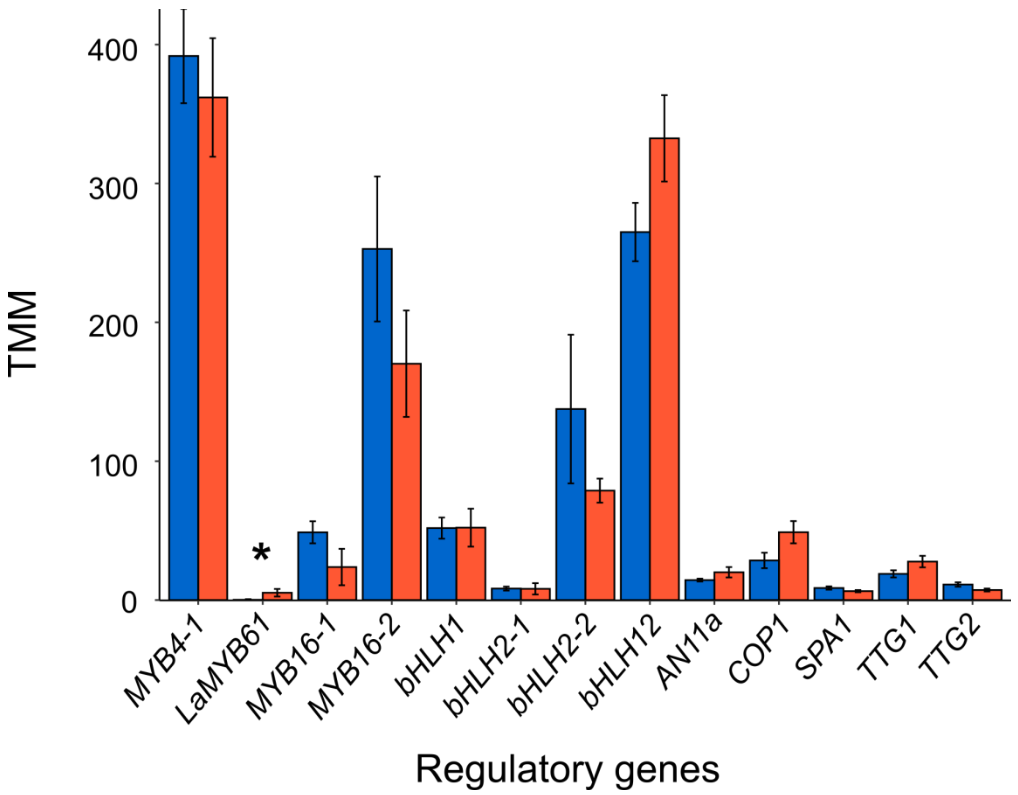

Supplementary Figure 3. Expression of regulatory genes involved in the flavonoid biosynthetic pathway of *L. arvensis*. The colored bars represent the average expression level in TMM (trimmed mean of M-values) units and error bars show standard deviation. Regulatory gene with differential expression between flower colors is indicated (*).

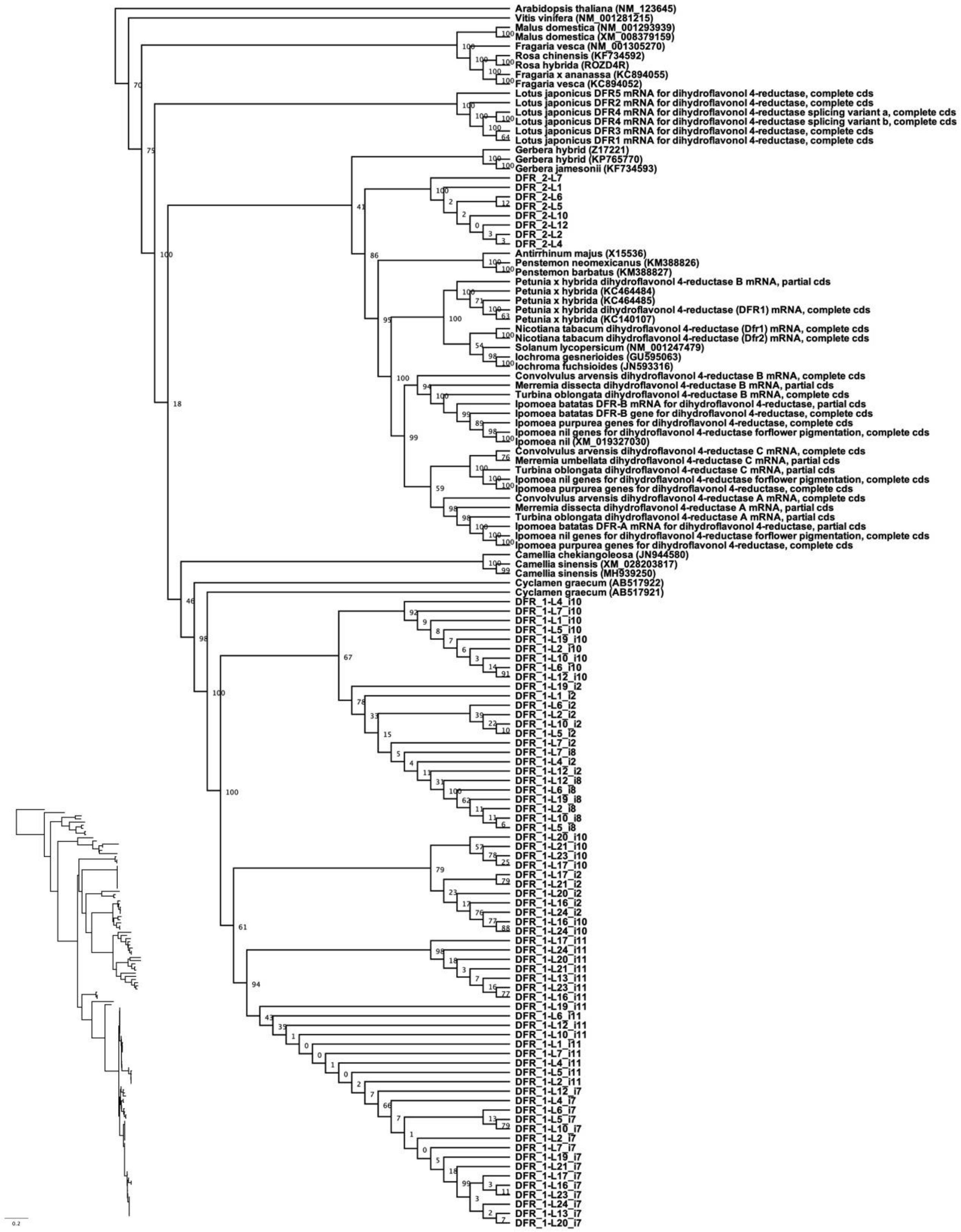

Supplementary Figure 4. Maximum likelihood phylogenetic analysis of *DFR-1* and *DFR-2* coding sequences for all *L. arvensis* isoforms (L1-12 = orange; L13-24 = blue). We used the top BLASTn hits (scientific name followed by followed by Genbank Accession numbers) and additional Genbank sequences from species with known *DFR* variation (e.g. Des Marais and Rausher, 2008) as references to confirm *DFR-1* and *DFR-2* diversification in Supplemental Figure 2. Bootstrap values are provided at the node. We removed the 5’ and 3’ ends (24bp and 52bp, respectively) of the coding sequence because of alignments ambiguities.
