# Supplementary material for: Changes at a Critical Branchpoint in the Anthocyanin Biosynthetic Pathway Underlie the Blue to Orange Flower Color Transition in Lysimachia arvensis
Source: Front Plant Sci. 2021 Feb 22;12:633979. doi: 10.3389/fpls.2021.633979 (PMC7937975; doi:10.3389/fpls.2021.633979)
Supplement: Supplementary file 1 [file Data_Sheet_1.zip › Supplementary Tables.pdf]

Supplementary Table 1. Total number of flavonoid biosynthetic pathway structural genes studied.

Gene abbreviation	Gene name	Kegg orthology code	Detected in *L.arvensis*
-	*2'-hydroxyisoflavone reductase*	K05281	yes
3AT	*Anthocyanidin 3-O-glucoside 6''-O-acyltransferase*	K21383	no
3GGT	*Anthocyanidin 3-O-glucoside 2''-O-glucosyltransferase*	K12933	yes
3GT	*Anthocyanin 3'-O-beta-glucosyltransferase*	K12939	yes
3MaT1	*Anthocyanin 3-O-glucoside-6''-O-malonyltransferase*	K12931	no
3MaT2	*Anthocyanidin 3-O-glucoside-3'',6''-O-dimalonyltransferase*	K12932	no
4CL	*4-coumarate coa ligase*	K01904	yes
5AT	*Anthocyanin 5-aromatic acyltransferase*	K12936	yes
5MaT1	*Anthocyanin 5-O-glucoside-6'''-O-malonyltransferase*	K12934	no
5MaT2	*Anthocyanin 5-O-glucoside-4'''-O-malonyltransferase*	K12935	no
7-IOMT	*Isoflavone 7-O-methyltransferase*	K13262	no
AIMT1	*Trans-anol O-methyltransferase*	K17059	no
ANR	*Anthocyanidin reductase*	K08695	yes
ANS	*Anthocyanidin synthase*	K05277	yes
AOMT	*Flavonoid O-methyltransferase*	K13272	no
AS1	*Aureusidin synthase*	K13079	yes
ATTSM1	*Caffeoyl coa 3-O-methyltransferase*	K13067	yes
Beta-glucosidase	*Beta-glucosidase*	K01188	yes
BRT1	*Sinapate 1-glucosyltransferase*	K13068	yes
BZ1	*Anthocyanidin 3-O-glucosyltransferase*	K12930	yes
C12RT1	*Flavanone 7-O-glucoside 2''-O-beta-L-rhamnosyltransferase*	K13080	no
-	*Chalcone 4'-O-glucosyltransferase*	K21372	no
C4h	*Cinnamate 4-hydroxylase*	-	yes
CAD	*Cinnamyl alcohol dehydrogenase*	K00083	yes
Caffeoyl-coa	*Caffeoyl coa O-methyltransferase*	K00588	yes
CCR	*Cinnamoyl coa reductase*	K09753	yes
CFAT	*Coniferyl alcohol acyltransferase*	K17054	no
CHI	*Chalcone isomerase*	K01859	yes
CHS	*Chalcone synthase*	K00660	yes
COMT	*Caffeic acid 3-O-methyltransferase*	K13066	yes
CSE	*Caffeoylshikimate esterase*	K18368	yes
CVOMT1	*Chavicol O-methyltransferase*	K17058	no
CYP71D9, F6H	*Flavonoid 6-hydroxylase*	K13267	no
CYP73A	*Trans-cinnamate 4-monooxygenase*	K00487	no
CYP75B1	*Flavonoid 3'-monooxygenase*	K05280	no
CYP81E1_7	*Isoflavone 2'-hydroxylase*	K13260	yes
CYP81E9	*Isoflavone 3'-hydroxylase*	K22932	yes
CYP84A; F5H	*Ferulate-5-hydroxylase*	K09755	yes
CYP93A1	*3,9-dihydroxypterocarpan 6a-monooxygenase*	K13261	yes
CYP93B2_16	*Flavone synthase II*	K23179	no
CYP93C	*2-hydroxyisoflavanone synthase*	K13257	no
CYP98A8	*Cytochrome P450 family 98 subfamily A polypeptide 8*	K15506	no
CYP98A9	*Cytochrome P450 family 98 subfamily A polypeptide 9*	K23662	yes
CYP98A, C3'H	*5-O-(4-coumaroyl)-D-quinate 3'-monooxygenase*	K09754	no
DFR	*Dihydroflavonol 4 reductase*	K13082	yes
DICGT	*Chalcononaringenin 2'-O-glucosyltransferase*	K13078	yes
EGS1	*Eugenol synthase*	K17055	no
EOMT1	*Eugenol O-methyltransferase*	K17058	no
F3'5'H	*Flavonoid 3',5'-hydroxylase*	K13083	yes
F3H	*Flavanone 3-dioxygenase*	K00475	yes
F3'H	*Flavonoid 3'-hydroxylase*	-	yes
Feruloyl-coa	*Feruloyl-CoA 6-hydroxylase*	K06892	yes
FG3	*Flavonol 3-O-glucoside*	K22794	no
-	*Flavonol 3-O-glucosyltransferase*	K10757	yes
-	*Flavonol 3-O-methyltransferase*	K05279	yes

-	*Flavonol 3-sulfotransferase*	K13270	yes
-	*Flavonol 4'-sulfotransferase*	K13271	yes
FLS	*Flavonol synthase*	K05278	yes
FNSI	*Flavone synthase I*	K13077	no
GT1	*Anthocyanidin 5,3-O-glucosyltransferase*	-	yes
HCT	*Shikimate o-hydroxycinnamoyltransferase*	K13065	yes
HI4OMT	*Isoflavone 4'-O-methyltransferase*	K13259	no
HIDH	*2-hydroxyisoflavanone dehydratase*	K13258	no
HMM	*(+)-6a-hydroxymaackiain 3-O-methyltransferase*	K21513	no
IEMT1	*(Iso)eugenol O-methyltransferase*	K17057	yes
IF7GT	*Isoflavone 7-O-glucosyltransferase*	K13263	no
IF7MAT	*Isoflavone 7-O-glucoside-6''-O-malonyltransferase*	K13264	yes
IGS1	*Isoeugenol synthase 1*	K17056	yes
-	*Kaempferol 3-O-beta-D-galactosyltransferase*	K13269	yes
katG	*Catalase peroxidase*	K03782	no
LAR	*Leucoanthocyanidin reductase*	K13081	yes
NOMT	*Naringenin 7-O-methyltransferase*	K22440	no
OMT	*O-methyltransferase domain*	-	yes
OMT1	*Desmethylxanthohumol 6'-O-methyltransferase*	K21581	no
OMT2	*Xanthohumol 4'-O-methyltransferase*	K21582	yes
PAL	*Phenylalanine ammonia-lyase*	K10775	yes
Peroxidase	*Peroxidase*	K00430	yes
PKR	*Polyketide reductase*	K08243	yes
PRDX6	*Peroxiredoxin 6, 1-Cys peroxiredoxin*	K11188	yes
PTR	*Pterocarpan reductase*	K13266	yes
REF1	*Coniferyl-aldehyde dehydrogenase*	K12355	yes
SCPL19, SNG2	*Serine carboxypeptidase-like 19*	K09756	no
SCPL8, SNG1	*Serine carboxypeptidase-like 8*	K09757	no
SOMT-2	*Flavonoid 4'-O-methyltransferase*	K23053	no
TOGT1	*Scopoletin glucosyltransferase*	K23260	no
UF3GT	*Udp glucose: flavonoid 3-o-glucosyltransferase*	-	yes
UGT72E	*Coniferyl alcohol glucosyltransferase*	K12356	yes
UGT73C6	*Flavonol 3-O-L-rhamnoside-7-O-glucosyltransferase*	K22771	yes
UGT75C1	*Anthocyanidin 3-O-glucoside 5-O-glucosyltransferase*	K12338	yes
UGT78D1	*Flavonol 3-O-rhamnosyltransferase*	K15787	no
UGT79B1	*Anthocyanidin 3-O-glucoside 2'''-O-xylosyltransferase*	K17193	yes
uidA; GUSB	*Beta-glucuronidase*	K01195	yes
VR	*Vestitone reductase*	K13265	yes

Supplementary Table 2. Flavonoid biosynthetic pathway regulatory genes studied. A total of 46 regulatory genes involved in flavonoid biosynthetic pathway were detected in literature.

Gene family	Gene abbreviation	Gene name	References	Detected in *L.arvensis*
**R2R3-MYB**	*MYB16; MIXTA*	*Transcription factor myb16*	Noda et al., 1994	Yes
	MYB4	*Transcription repressor myb4*	Dubos et al., 2010; Li, 2014; Zheng et al., 2020	Yes
	TT2; MYB123	*Transparent testa 2*	Dubos et al., 2010; Li, 2014; Xu et al., 2015	No
	PAP1; MYB75; SIAA1	*Production of anthocyanin pigment 1*	Dubos et al., 2010; Li, 2014; Xu et al., 2015	No
	PAP2; MYB90	*Production of anthocyanin pigment 2*	Dubos et al., 2010; Li, 2014; Xu et al., 2015	No
	PAP4; MYB114	*Production of anthocyanin pigment 4*	Dubos et al., 2010; Li, 2014; Xu et al., 2015	No
	MYB66; WER	*Transcription factor MYB66*	Li, 2014; Xu et al., 2015	No
	MYB23	*Transcription factor MYB23*	Xu et al., 2015	No
	MYB5	*Transcription repressor MYB5*	Dubos et al., 2010; Hichri et al., 2011; Xu et al., 2015; Zheng et al., 2020	No
	MYB82	*Transcription factor MYB82*	Liang et al., 2014	No
	AN2	*Anthocyanin 2*	De Vetten et al., 1997; Hichri et al., 2011; Gates et al., 2017	No
	MYB27	*Transcription factor MYB27*	Gates et al., 2017	No
	MYBx	*R3-MYB anthocyanin repressor*	Li, 2014; Gates et al., 2017	No
	CPC	*Caprice*	Hichri et al., 2011; Li, 2014; Gates et al., 2017	No
	MYBL1	*MYB-like 1*	Gates et al., 2017	No
	MYBL2	*MYB-like 2*	Li, 2014; Xu et al., 2015; Gates et al., 2017	No
	MYB113	*Transcription factor MYB113*	Dubos et al., 2010; Li, 2014	No
	ETC1	*Enhancer of TRY and CPC 1*	Lepiniec et al., 2006; Li, 2014	No
	ETC2	*Enhancer of TRY and CPC 2*	Lepiniec et al., 2006; Li, 2014	No
	TRY	*Triptychon*	Hichri et al., 2011; Li, 2014	No
	MYB0; GL1	*Glabra 1*	Li, 2014; Xu et al., 2015	No
	MYB12; PFG1	*Production of flavonol glycosides 1*	Dubos et al., 2010; Hichri et al., 2011; Xu et al., 2015	No
	MYB11; PFG2	*Production of flavonol glycosides 2*	Dubos et al., 2010; Hichri et al., 2011; Xu et al., 2015	No
	MYB111; PFG3	*Production of flavonol glycosides 3*	Dubos et al., 2010; Hichri et al., 2011; Xu et al., 2015	No
	MYB7; ATY49	*Transcription factor MYB7*	Dubos et al., 2010	No
	MYB2	*Transcription factor MYB2*	Feng et al., 2020	No
**bHLH**	*bHLH1; GL3; MYC6*	*Basic helix loop helix 1*	Hichri et al., 2011; Li, 2014; Xu et al., 2015; Feng et al., 2020	Yes
	bHLH12; MYC1	*Basic helix loop helix 12*	Hichri et al., 2011	Yes
	bHLH2; EGL1	*Basic helix loop helix 2*	Hichri et al., 2011	Yes
	JAF13	*Transcription factor jaf13*	Hichri et al., 2011; Gates et al., 2017	No
	AN1	*Anthocyanin 1*	Hichri et al., 2011; Li, 2014; Gates et al., 2017	No
	bHLH42; TT8	*transparent testa 8*	Hichri et al., 2011; Li, 2014; Xu et al., 2015	No
	Lc	*Anthocyanin regulatory Lc protein*	Fan et al., 2016	No
	R-S	*Anthocyanin regulatory R-S protein*	Cone et al., 1993	No
	C1	*Anthocyanin regulatory C1 protein*	Dubos et al., 2010; Hichri et al., 2011	No
**WDR**	*AN11a*	*Wd repeat containing protein lwd1*	De Vetten et al., 1997; Hichri et al., 2011; Gates et al., 2018	Yes
	AN11b	*Wd repeat containing protein lwd2*	De Vetten et al., 1997; Hichri et al., 2011; Gates et al., 2018	No
	COP1	*E3 ubiquitin protein ligase cop1*	Xu et al., 2015	Yes
	SPA1	*Protein suppressor of phya 105 1*	Xu et al., 2015	Yes
	TTG1	*Transparent testa glabra 1*	Tsuchiya et al., 2004; Hichri et al., 2011; Li, 2014; Xu et al., 2015	Yes
**WRKY**	*TTG2; DSL1; WRKY44*	*Transparent testa glabra 2*	Hichri et al., 2011; Xu et al., 2015	Yes
**Other**	*ANL2*	*Anthocyaninless 2*	Xu et al., 2015	No
	FUS3; FUSCA3	*B3 domain-containing transcription factor FUS3*	Tsuchiya et al., 2004	No

UPL3; KAKTUS	*Ubiquitin-protein ligase UPL3*	Xu et al., 2015	No
TT1; WIP1; TTL1	*Transparent testa 1*	Li, 2014; Xu et al., 2015	No
TT16; ABS; AGL32	*Transparent testa 16*	Hichri et al., 2011; Li, 2014; Xu et al., 2015	No

References:

Cone, K. C., Cocciolone, S. M., Burr, F. A., and Burr, B. (1993). Maize anthocyanin regulatory gene pl is a duplicate of c1 that functions in the plant. *Plant Cell* 5:1795. doi: 10.2307/3869695

De Vetten, N., Quattrocchio, F., Mol, J., and Koes, R. (1997). The an11 locus controlling flower pigmentation in petunia encodes a novel WD-repeat protein conserved in yeast, plants, and animals. *Genes Dev.* 11, 1422–1434.doi: 10.1101/gad.11.11.1422

Dubos, C., Stracke, R., Grotewold, E., Weisshaar, B., Martin, C., and Lepiniec, L. (2010). MYB transcription factors in *Arabidopsis. Trends Plant Sci.* 15, 573–581. doi: 10.1016/j.tplants.2010.06.005

Fan, X., Fan, B., Wang, Y., and Yang, W. (2016). Anthocyanin accumulation enhanced in Lc-transgenic cotton under light and increased resistance to bollworm. *Plant Biotechnol. Rep.* 10, 1–11.doi: 10.1007/s11816-015-0382-3

Feng, C., Ding, D., Feng, C., and Kang, M. (2020). The identification of an R2R3-MYB transcription factor involved in regulating anthocyanin biosynthesis in *Primulina swinglei* flowers. Gene 752:144788. doi: 10.1016/j.gene.2020.144788

Gates, D. J., Olson, B. J. S. C., Clemente, T. E., and Smith, S. D. (2017). A novel R3 MYB transcriptional repressor associated with the loss of floral pigmentation in *Iochroma. New Phytol.* 217, 1346–1356.doi: 10.1111/nph.14830

Hichri, I., Barrieu, F., Bogs, J., Kappel, C., Delrot, S., and Lauvergeat, V. (2011). Recent advances in the transcriptional regulation of the flavonoid biosynthetic pathway. *J. Exp. Bot.* 62, 2465–2483.doi: 10.1093/jxb/erq442

Lepiniec, L., Debeaujon, I., Routaboul, J.-M., Baudry, A., Pourcel, L., Nesi, N., et al. (2006). Genetics and biochemistry of seed flavonoids. *Annu. Rev. Plant Biol.* 57, 405–430. doi: 10.1146/annurev.arplant.57.032905.105252

Li, S. (2014). Transcriptional control of flavonoid biosynthesis. *Plant Signal Behav.* 9:e27522. doi: 10.4161/psb.27522

Liang, G., He, H., Li, Y., Ai, Q., and Yu, D. (2014). MYB82 functions in regulation of trichome development in *Arabidopsis*. *J. Exp. Bot.* 65, 3215–3223. doi: 10.1093/jxb/eru179

Noda, K., Glover, B. J., Linstead, P., and Martin, C. (1994). Flower colour intensity depends on specialized cell shape controlled by a Myb-related transcription factor. Nature 369, 661–664. doi: 10.1038/369661a0

Tsuchiya, Y., Nambara, E., Naito, S., and McCourt, P. (2004). The FUS3 transcription factor functions through the epidermal regulator *TTG1* during embryogenesis in *Arabidopsis*. *Plant J.* 37, 73–81. doi: 10.1046/j.1365-313x.2003.01939.x

Xu, W., Dubos, C., and Lepiniec, L. (2015). Transcriptional control of flavonoid biosynthesis by MYB–bHLH–WDR complexes. *Trends Plant Sci.* 20, 176–185. doi: 10.1016/j.tplants.2014.12.00

Zheng, X., Om, K., Stanton, K. A., Thomas, D., Cheng, P. A., Eggert, A., et al. (2020). MYB5a/NEGAN activates petal anthocyanin pigmentation and shapes the MBW regulatory network in *Mimulus luteus var. variegatus*. *bioRxiv* [Preprint]. doi: 10.1101/2020.04.09.030536

Supplementary Table 3. Flavonoid standards for UHPLC-MS analysis. Standards used for identifying and quantifying flavonoids in the UHPLC-MS analysis, their mass transitions, and the references in which the compound derivatives were identified in *L. arvensis*.

Flavonoid type	Standard	Mass transitions	References
Anthocyanin	Malvidin	331.66/331.57	Lawrence et al. 1939, Harborne 1968, Ishikura 1981, Kawashty et al. 1998, Freyre & Griesbach 2004
Anthocyanin	Pelargonidin	271.02/271.02	Lawrence et al. 1939, Harborne 1968, Kawashty et al. 1998, Freyre & Griesbach 2004
Anthocyanin	Cyanidin	287.08/287.07	-
Anthocyanin	Delphinidin	303.06/302.76	Harborne 1968, Freyre & Griesbach 2004
Flavonol	Kaempferol	286.93/287.61	Kawashty et al. 1998
Flavonol	Quercetin	164.65/303.35	Kawashty et al. 1998
Flavone	Luteolin	287.10/287.21	Ishikura 1981
Flavonol	Isorhamnetin	323.17/204.26	Kawashty et al. 1998

Supplementary Table 4. Summary of sequencing and assembly results for the 16 *L. arvensis* petal samples.

Sample ID	Flower color	Number of reads	Number of filtered reads	Reads aligned to the assembly (%)	Total number of bases aligned	Number of transcripts	Number of trinity genes*
**Lyar_1**	orange	26,918,730	21,577,770	98.19	3,102,351,362	150,034	41,202
**Lyar_2**	orange	30,544,193	24,341,034	98.04	3,511,917,285	153,036	39,046
**Lyar_4**	orange	28,356,639	22,933,654	98.23	3,311,802,072	147,321	37,758
**Lyar_5**	orange	31,542,891	25,291,784	98.24	3,642,359,970	156,240	39,346
**Lyar_6**	orange	32,912,856	26,662,938	98.25	3,834,908,823	160,116	40,872
**Lyar_7**	orange	30,718,374	23,802,707	97.95	3,539,104,413	155,366	40,684
**Lyar_10**	orange	29,353,348	23,836,262	98.11	3,436,734,803	151,084	37,790
**Lyar_12**	orange	29,060,571	23,552,368	98.35	3,390,018,830	148,293	38,361
**Lyar_13**	blue	31,348,555	25,548,362	98.31	3,672,433,439	161,757	40,909
**Lyar_16**	blue	26,079,191	21,032,716	98.17	3,034,751,021	148,713	39,359
**Lyar_17**	blue	26,238,711	21,194,645	98.20	3,053,229,548	150,024	39,482
**Lyar_19**	blue	37,348,756	30,482,778	98.30	4,387,462,342	193,216	42,243
**Lyar_20**	blue	33,610,203	27,363,548	98.30	4,069,453,932	154,142	37,166
**Lyar_21**	blue	31,594,397	25,750,601	98.35	3,830,424,801	156,441	38,792
**Lyar_23**	blue	26,679,503	21,744,545	98.28	3,229,827,114	146,776	37,948
**Lyar_24**	blue	49,814,154	40,996,370	98.40	5,918,376,202	178,648	38,739

* Trinity genes identified as having transcripts per million values above 1.

Supplementary Table 5. Flavonoid biosynthetic pathway structural genes detected in petals of *L. arvensis*.

Gene abbreviation	Gene name	No. trinity genes (or gene copies?)
-	*2'-hydroxyisoflavone reductase*	1
3GGT	*Anthocyanidin 3-O-glucoside-2''-o-glucosyltransferase*	1
3GT	*Anthocyanin 3'-O-beta-glucosyltransferase*	4
4CL	*4-coumarate coa ligase*	3
5AT	*Anthocyanin 5-aromatic-acyltransferase*	2
ANR	*Anthocyanidin reductase*	1
ANS	*Anthocyanidin synthase*	1
AS1	*Aureusidin synthase*	1
ATTSM1	*Caffeoyl coa-3-O-methyltransferase*	1
Beta-glucosidase	*Beta-glucosidase*	17
BRT1	*Sinapate 1-glucosyltransferase*	2
BZ1	*Anthocyanidin 3-O-glucosyltransferase*	4
C4h	*Cinnamate 4-hydroxylase*	2
CAD	*Cinnamyl alcohol dehydrogenase*	2
Caffeoyl-coa	*Caffeoyl coa O-methyltransferase*	3
CCR	*Cinnamoyl coa reductase*	5
CHI	*Chalcone isomerase*	2
CHS	*Chalcone synthase*	1
COMT	*Caffeic acid 3-O-methyltransferase*	1
CSE	*Caffeoylshikimate esterase*	7
CYP81E1_7	*Isoflavone 2'-hydroxylase*	2
CYP81E9	*Isoflavone 3'-hydroxylase*	1
CYP84A	*Ferulate 5-hydroxylase*	5
CYP93A1	*39-dihydroxypterocarpan-6a-monooxygenase*	1
CYP98A9	*Cytochrome P450 family 98 subfamily A polypeptide 9*	1
DFR	*Dihydroflavonol 4-reductase*	4
DICGT	*Chalcononaringenin 2'-o-glucosyltransferase*	2
F3'5'H	*Flavonoid 3'5'-hydroxylase*	4
F3H	*Flavanone 3-dioxygenase*	1
F3'H	*Flavonoid 3'-hydroxylase*	3
Feruloyl-coa	*Feruloyl coa 6'-hydroxylase-2*	2
-	*Flavonol 3-O-glucosyltransferase*	1
-	*Flavonol 3-O-methyltransferase*	5
-	*Flavonol 3-sulfotransferase*	2
-	*Flavonol 4'-sulfotransferase*	1
FLS	*Flavonol synthase*	2
GT1	*Anthocyanidin 53-O-glucosyltransferase*	1
HCT	*Shikimate O-hydroxycinnamoyltransferase*	1
IEMT1	*Eugenol O-methyltransferase*	5
IF7MAT	*Malonyl coa: isoflavone 7-o-glucoside-6''-O*	1
IGS1	*Isoeugenol synthase 1*	1
-	*Kaempferol 3-O-beta-d-galactosyltransferase*	1
LAR	*Leucoanthocyanidin reductase*	1
OMT	*O-methyltransferase*	3
OMT2	*Xanthohumol 4'-o-methyltransferase*	2
PAL	*Phenylalanine ammonia lyase*	1
Peroxidase	*Peroxidase*	5
PKR	*Polyketide reductase*	4
PRDX6	*Peroxiredoxin 6-1-cys-peroxiredoxin*	1
PTR	*Pterocarpan reductase*	2
REF1	*Coniferyl aldehyde dehydrogenase*	2
UF3GT	*Udp-glucose: flavonoid 3-o-glucosyltransferase*	5
UGT72E	*Coniferyl alcohol glucosyltransferase*	5
UGT73C6	*Flavonol 3-O-L-rhamnoside-7-O-glucosyltransferase*	2

UGT75C1	*Anthocyanidin 3-O-glucoside-5-o-glucosyltransferase*	4
UGT79B1	*Anthocyanidin 3-O-glucoside-2'''-o-xylosyltransferase*	1
uidA; GUSB	*Beta-glucuronidase*	5
VR	*Vestitone reductase*	2

Supplementary Table 6. Flavonoid biosynthetic pathway regulatory genes detected in petals of *L. arvensis*.

Regulatory gene family	Gene abbreviation	Gene name	No. trinity genes
**R2R3-MYB**	*MYB16; MIXTA*	*Transcription factor myb16*	2
	MYB4	*Transcription repressor myb4*	2
**bHLH**	*bHLH1; GL3; MYC6*	*Basic helix loop helix 1*	1
	bHLH12; MYC1	*Basic helix loop helix 12*	1
	bHLH2; EGL1	*Basic helix loop helix 2*	2
**WDR**	*AN11a*	*Wd repeat containing protein lwd1*	1
	COP1	*E3 ubiquitin protein ligase cop1*	1
	SPA1	*Protein suppressor of phya 105 1*	1
	TTG1	*Transparent testa glabra 1*	1
**WRKY**	*TTG2; DSL1; WRKY44*	*Transparent testa glabra 2*	1

Supplementary Table 7. *F3'5'H* synonymous and non-synonymous SNP frequencies between blue and orange isotigs of the CDS, 5' UTR and 3' UTR sequences.

**Site no. (bp)**	**Reference base**	**Alternate base**	**Blue frequency**	**Orange frequency**	**Frequency difference (blue – orange)**
**CDS**					
6*	A	T	1	0.17	0.83
222*	A	T	1	0.17	0.83
409	C	T	0.57	0	0.57
435	G	A	0.57	0	0.57
472*	C	A	0.5	0	0.5
615	T	C	0.43	0	0.43
657	T	G	0.36	0	0.36
669	C	T	0.32	0	0.32
864	A	G	0.5	0	0.5
882	C	T	1	0.33	0.67
996	G	A	0.61	0	0.61
1,017	A	C	0.61	0	0.61
1,044	T	C	0.5	0	0.5
1,050	C	G	0.5	0.13	0.38
1,149	T	C	0.5	0	0.5
1,194	C	T	1	0.91	0.09
1,347	G	A	0.5	0	0.5
1,374	C	T	0.5	0	0.5
1,377	A	T	0.5	0	0.5
1,426*	A	G	0.64	0	0.64
1,467	G	A	0.64	0	0.64
1,488	C	T	0.64	0	0.64
**5' UTR**					
(-)118	A	T	0.79	0	0.79
(-)56	T	C	0.79	0	0.79
(-)42	A	T	0.79	0	0.79
(-)8	G	A	1	0.91	0.09
(-)4	A	T	1	0.91	0.09
**3' UTR**					
(+)4	T	A	0.64	0	0.64
(+)8	A	T	0.64	0	0.64
(+)15	G	A	0.64	0	0.64
(+)19	G	A	0.64	0	0.64
(+)54	A	G	0.59	0	0.59
(+)90	T	C	0.52	0	0.52

Alignment of the CDS is used as reference.
Not including blue sample L19, which is closely related to orange samples.
As reference base was used the one with higher frequency in blue isotigs.
(-) sign is followed by the number of base pairs (bp) before the start codon.
(+) sign is followed by the number of base pairs (bp) after the stop codon.
* indicates non-synonymous SNPs.

Supplementary Table 8. *DFR-1* synonymous and non-synonymous SNP frequencies between blue and orange isotigs of the CDS sequences.

Site no. (bp)	Reference base	Alternate base	Blue frequency	Orange frequency	Frequency difference (blue – orange)
14*	T	C	0.59	0.16	0.43
16	T	C	1	0.88	0.12
117	T	A	1	0.22	0.78
204	G	A	1	0.32	0.68
291	T	A	1	0.59	0.41
300	A	G	0.38	0	0.38
303	T	G	0.38	0	0.38
312	A	G	0.92	0.41	0.51
321	C	T	0.29	0	0.29
324	G	T	0.25	0	0.25
327	T	C	0.21	0	0.21
328	T	C	0.21	0	0.21
330	T	C	0.78	0.41	0.37
333	T	C	0.25	0	0.25
339	T	A	0.17	0	0.17
342	A	C	0.17	0	0.17
358*	G	A	0.41	0	0.41
369	A	G	0.32	0	0.32
434*	A	G	0.91	0.41	0.50
441	T	C	0.39	0	0.39
462	T	C	1	0.41	0.59
465	T	C	1	0.41	0.59
474*	G	A	0.52	0	0.52
495	T	C	1	0.54	0.46
525	T	A	0.62	0	0.62
628	C	A	0.21	0	0.21
629	T	C	0.21	0	0.21
684	A	G	1	0.31	0.69
717	C	T	1	0	1.00
729	G	T	1	0.5	0.50
743*	A	C	1	0.5	0.50
754	C	A	0.77	0	0.77
828	C	T	1	0.5	0.50
840	T	C	1	0	1.00
852	C	T	1	0.28	0.72
858	T	C	1	0.75	0.25
859*	G	C	1	0	1.00
863*	G	A	1	0	1.00
864	C	T	1	0.75	0.25
873	C	T	1	0.75	0.25
885	A	G	1	0.75	0.25
891	C	T	1	0	1.00

899	A	G	1	0.75	0.25
905	T	C	1	0.75	0.25
912	C	T	1	0.81	0.19
913*	G	C	1	0.19	0.81
942	T	C	1	0.81	0.19
959*	A	C	1	0	1.00
976	C	T	1	0.84	0.16
978	C	G	0.67	0	0.67
984	G	A	1	0.84	0.16
985	C	T	1	0.84	0.16
1,001	C	T	1	0.78	0.22

Not including blue sample L19, which is closely related to orange samples.
As reference base was used the one with higher frequency in blue isotigs.
* indicates non-synonymous SNPs.
